# Analyses of Seven New Genomes of *Xanthomonas citri* pv. *aurantifolii* Strains, Causative Agents of Citrus Canker B and C, Show a Reduced Repertoire of Pathogenicity-Related Genes

**DOI:** 10.3389/fmicb.2019.02361

**Published:** 2019-10-11

**Authors:** Natasha Peixoto Fonseca, José S. L. Patané, Alessandro M. Varani, Érica Barbosa Felestrino, Washington Luiz Caneschi, Angélica Bianchini Sanchez, Isabella Ferreira Cordeiro, Camila Gracyelle de Carvalho Lemes, Renata de Almeida Barbosa Assis, Camila Carrião Machado Garcia, José Belasque Jr., Joaquim Martins Jr., Agda Paula Facincani, Rafael Marini Ferreira, Fabrício José Jaciani, Nalvo Franco de Almeida, Jesus Aparecido Ferro, Leandro Marcio Moreira, João C. Setubal

**Affiliations:** ^1^Programa de Pós-graduação em Biotecnologia, Núcleo de Pesquisas em Ciências Biológicas, Universidade Federal de Ouro Preto, Ouro Preto, Brazil; ^2^Laboratório Especial de Ciclo Celular, Instituto Butantan, São Paulo, Brazil; ^3^Departamento de Tecnologia, Universidade Estadual Paulista, UNESP, Campus de Jaboticabal, Jaboticabal, Brazil; ^4^Departamento de Fitopatologia e Nematologia, Escola Superior de Agricultura Luiz de Queiroz, Universidade de São Paulo, Piracicaba, Brazil; ^5^Departamento de Bioquímica, Instituto de Química, Universidade de São Paulo, São Paulo, Brazil; ^6^Fundo de Defesa da Citricultura (FUNDECITRUS), São Paulo, Brazil; ^7^Faculdade de Computação, Universidade Federal de Mato Grosso do Sul, Campo Grande, Brazil; ^8^Departamento de Ciências Biológicas, Instituto de Ciências Exatas e Biológicas, Universidade Federal de Ouro Preto, Ouro Preto, Brazil

**Keywords:** effectors, adaptation, virulence, *Xanthomonas* evolution, genome sequencing

## Abstract

*Xanthomonas citri* pv. *aurantifolii* pathotype B (XauB) and pathotype C (XauC) are the causative agents respectively of citrus canker B and C, diseases of citrus plants related to the better-known citrus canker A, caused by *Xanthomonas citri* pv. *citri*. The study of the genomes of strains of these related bacterial species has the potential to bring new understanding to the molecular basis of citrus canker as well as their evolutionary history. Up to now only one genome sequence of XauB and only one genome sequence of XauC have been available, both in draft status. Here we present two new genome sequences of XauB (both complete) and five new genome sequences of XauC (two complete). A phylogenomic analysis of these seven genome sequences along with 24 other related *Xanthomonas* genomes showed that there are two distinct and well-supported major clades, the XauB and XauC clade and the *Xanthomonas citri* pv. *citri* clade. An analysis of 62 Type III Secretion System effector genes showed that there are 42 effectors with variable presence/absence or pseudogene status among the 31 genomes analyzed. A comparative analysis of secretion-system and surface-structure genes showed that the XauB and XauC genomes lack several key genes in pathogenicity-related subsystems. These subsystems, the Types I and IV Secretion Systems, and the Type IV pilus, therefore emerge as important ones in helping explain the aggressiveness of the A type of citrus canker and the apparent dominance in the field of the corresponding strain over the B and C strains.

## Introduction

Citrus is an important worldwide crop (FAS, 2019) that has been threatened by various diseases over many decades. Even though citrus Huanglongbin (greening) is today the major citrus threat (Wang et al., 2017), citrus canker (Goto, 1992; Schubert and Miller, 1996; Schubert et al., 2001) still is an important disease (CAB-International, 2019), especially in Brazil (Mendonça et al., 2017).

Three species of bacteria of the genus *Xanthomonas* are associated with citrus canker diseases in citrus: *Xanthomonas citri* subsp. *citri* (Xcc) pathotypes A, A^∗^ and A^w^, *X. citri* subsp. *aurantifolii* pathotypes B and C (XauB and XauC), and *X. alfalfae* subsp. *citrumelonis* (Xacm). Xcc, XauB and XauC are respectively the causative agents of citrus canker A, B, and C, which cause small necrotic raised lesions surrounded by a water-soaked margin (Civerolo, 1984). Citrus canker A, the most aggressive, remains a concern in all citrus growing regions in Asia and South America (CAB-International, 2019). XauB strains are less aggressive, and XauC strains have a more restricted host range, when compared with symptoms and host range of Xcc, respectively. Canker B is currently known to be present only in Argentina, Paraguay, and Uruguay (Civerolo, 1984); moreover, XauB may have been eradicated even from this restricted region by competition from Xcc (Chiesa et al., 2013). Canker C is limited to the state of São Paulo, Brazil (Malavolta Júnior et al., 1984); the most recent field report dates to 2009 (Jaciani et al., 2009). Xacm is the causal agent of citrus bacterial spot, which induces symptoms very similar to those of canker, but the lesions are flat and not raised.

Sequencing of the *X. citri* susbsp. *citri* strain A306 genome (A306) allowed the characterization of important properties of this more aggressive pathotype (da Silva et al., 2002). Following that work, genomes of the other pathotypes were sequenced and compared with each other (Jalan et al., 2013; Bodnar et al., 2017).

Given the phylogenetic relatedness of the causal pathogens of cankers A, B, and C, the comparative study of XauB and XauC strains at the genomic level offers the opportunity of achieving a better understanding of citrus canker disease in general. Up to now, only one XauB and only one XauC strain genome have been sequenced (Moreira et al., 2010). We therefore decided to sequence the genomes of additional strains of XauB and XauC. The newly sequenced isolates were selected because they showed significant differences in pathogenicity and aggressiveness when inoculated in different citrus genotypes and/or had different genetic characteristics (Jaciani, 2012; Table 1). The isolates XauB 1561 and XauB 1566 showed less virulence with respect to the other isolates and absence of clear infection symptoms, suggesting a probable loss of pathogenicity, besides being genetically different by AFLP and ERIC-PCR (Jaciani, 2012). The selection of XauC strains was based on the ability of some isolates to produce dark pigment when cultivated in NB or NA culture media (NB: 0.5% peptone, 0.3% beef extract; NA: 0.5% peptone, 0.3% beef extract, 1.5% agar), also observed in *X. citri* pv. *fuscans* and *X. campestris* pv. *vignicola* (Schaad et al., 2005; Schaad et al., 2006). XauC 535 and XauC 1609 cause lesions only in Mexican lime [*C. aurantifolia* (Christm.) Swingle] and Swingle citrumelo [*C. paradisi* Macfad. × *Poncirus trifoliata* (L.) Raf.], and in both hosts with mild symptoms. XauC 1609 was found to infect Swingle citrumelo under natural conditions (Jaciani et al., 2009), despite the fact that prior work suggested that only Mexican lime was susceptible to XauC (Malavolta Júnior et al., 1984). Additionally, XauC 535 and XauC 1609 were also differentiated by AFLP and BOX-PCR (Jaciani, 2012). The isolates XauC 763, XauC 867, and XauC 1559, which do not produce pigment, were distinguished in terms of pathogenicity and aggressiveness. XauC 763 and XauC 1559, which are also Mexican lime pathogens, caused injuries in Swingle citrumelo and Cravo mandarin (*C. reticulata* Blanco), and when inoculated in high concentration they infected Rangpur lime (*C. limonia* Osbeck), Persian lime [*C. latifolia* (Yu. Tanaka) Tanaka], lemon [*Citrus limon* (L.) Burm. f.], Grapefruit (*C. paradisi* Macfad.), and Cleopatra mandarin (*C. reshni* hort. ex Tanaka) (Jaciani, 2012). Finally, XauC 1559 was slightly more aggressive than XauC 763 when inoculated in Cravo mandarin, and XauC 867 presented a slightly more restricted pathogenicity and lower aggressiveness in Mexican lime compared to XauC 763 and XauC 1559 (Jaciani, 2012).

**TABLE 1:** Phenotype characteristics of eight *Xanthomonas citri* strains (Jaciani, 2012).

**Strain (short name)**	**Year**	**Origin**	**Pathogenicity on: Host**	**Pigment production**	**Swingle citrumelo**	**Hamlin sweet orange**	**Mexican lime**	**Persian lime**	**Rangpur lime**	**Lemon**	**Grapefruit**	**Cleopatra mandarin**	**Cravo mandarin**	**Ponkan mandarin**
XauB 1561	1981	Argentina	Lemon	No	−	−	±	−	−	−	−	−	−	±
XauB 1566	1990	Argentina	Lemon	No	+	−	−	−	−	−	±	−	−	−
XauC 535	2000	Brazil	Mexican lime	Yes	+	+	++	−	−	−	−	−	+	±
XauC 763	1981	Brazil	Mexican lime	No	++	±	+++	±	−	±	±	±	+	±
XauC 867	2002	Brazil	Mexican lime	No	++	±	+	±	−	−	−	±	±	±
XauC 1559	1981	Brazil	Mexican lime	No	++	±	+++	±	−	±	±	±	++	+
XauC 1609	2009	Brazil	Swingle citrumelo	Yes	+	±	+	−	−	−	−	−	±	−
Xac306 or A306	1997	Brazil	Sweet orange	No	++++	+++	++++	+++	++++	++	+++	++	+++	+++

**−, non-pathogenic (absence of symptom); ±, not aggressive (slight eruption at the wound site and no necrosis or water-soaking); +, weakly aggressive (limited necrosis and no water-soaking); ++, moderately aggressive (small necrosis and limited water-soaking); +++, aggressive (large necrosis surrounded by a water-soaking margin); ++++, highly aggressive (extensive necrosis surrounded by a water-soaking margin).**

Altogether, based on the information above, we have sequenced two new XauB and five new XauC genomes, with the aim of achieving a better understanding of the genomic basis of citrus canker and the evolutionary history of these strains. Together with 24 other public and closely related genomes, this allowed us to carry out a phylogenomic analysis as well as an investigation of selected gene families relevant in bacteria-plant interactions in general and in citrus canker in particular (Ryan et al., 2011), which we present here.

A note on taxonomic nomenclature: *Xanthomonas* species that are pathogenic to citrus were described in this study using names as proposed by Schaad et al. (2006), since this classification is adopted for all cited references found until the present. The other *Xanthomonas* species were described as proposed by Bui Thi Ngoc et al. (2010) and Constantin et al. (2016).

## Materials and Methods

### Media and Culture Conditions

The new genomes presented here were sequenced from strains stored both in autoclaved tap water at room temperature and at −80°C in NB medium (3 g/L meat extract, 5 g/L peptone) containing 25% glycerol. Each one of the strains was recovered from a −80°C stock, streaked on solid NA medium (3 g/L meat extract, 5 g/L peptone and 15g/L agar) and cultivated for 48 h at 29°C. For each strain, colonies were inoculated into 10 mL of liquid NB medium in a sterile 50 mL Falcon conical centrifuge tube and incubated at 29°C in a rotary shaker at 180 rpm for 16 h (final OD600 nm ∼1.0).

### DNA Extraction and Quantification

A volume of 2 mL of the culture was centrifuged at 16,000 g for 10 min at 4°C in a refrigerated benchtop microcentrifuge. The supernatant was discarded and the cell pellet was resuspended in 600 μL of Nuclei Lysis Solution supplied by Promega Wizard Genomic DNA purification kit (Promega Corporation, Madison, United States). Total DNA extraction was performed using Promega Wizard Genomic DNA purification kit according to manufacturer instructions. DNA quantity and quality were determined using NanoDrop ND-1000 spectrophotometer (NanoDrop Tech, Wilmington, DE, United States), Qubit 2.0 fluorometer (Invitrogen, Life Technologies, CA, United States) and 0.8% agarose gel electrophoresis. Each extraction yielded at least 5 μg of high-quality genomic DNA.

### Genome Sequencing and Assembly

The genomes of XauC 535, XauC 763, and XauC 867 strains were sequenced using the Illumina HiScanSQ platform at NGS Soluções Genômicas (Piracicaba, Brazil). An average of ∼20 M reads (2 × 100 bp) for each genome was generated. The raw reads were trimmed with seqyclean software^1^, using minimum phred value of 23, minimum read length of 30 bp, and removing custom Illumina TruSeq adapters. Genome assembly was carried out with SPAdes v3.8.1 (Bankevich et al., 2012) with default parameters. Contig sequences were assigned to plasmids using plasmidSPAdes v3.8.1 (Antipov et al., 2016).

The genomes of XauB1561, XauB1566, XauC1559, XauC1609 strains were sequenced using PacBio single molecule real-time (SMRT) technology at the Duke Center for Genomic and Computational Biology (United States). One SMRT library was sequenced for each sample using P6-C4 chemistry, generating reads with average length of 10–15 Kb, thus yielding ∼150X coverage of each genome. *De novo* assembly was conducted using SMRT^®^ Analysis Server v2.3.0^2^. Raw PacBio reads were mapped against the resulting contigs using the blasR aligner, and SNP corrections were conducted with variant-caller software using the quiver algorithm (both part of the Analysis Server).

The rationale for having some genomes sequenced using PacBio technology and some using Illumina technology was as follows. We wanted to ensure that we could provide complete genomes for both XauB and XauC, given that prior to this work only draft genomes were available for these pathotypes (Moreira et al., 2010). On the other hand our budget was limited, and we could afford PacBio sequencing for only four genomes. Under these constraints, the choice of which genomes to sequence by PacBio was arbitrary.

All assembled genomes were verified with CheckM (Parks et al., 2015), resulting in 100% completeness and 0% contamination for all of them.

### Genome Selection

For the purposes of phylogenomic analyses, we searched for genomes in NCBI/GenBank using “Xanthomonas citri” as a keyword, then looked at the automatic dendrogram generated by genomic distances on the NCBI website^3^, which reveals all genomes within this group, including all subspecies/lineages/varieties available as reference sequences (RefSeq). After downloading this tree, we searched for all different lineages, and then downloaded up to three genomes from each such lineage, if available, and preferentially (if possible) drawing from separate clades where the lineage appears in NCBI’s dendrogram, to avoid pseudoreplication (i.e., avoiding picking two closely related genomes). This led to a final dataset of 31 genomes.

### Phylogenomic Reconstruction

In order to generate comparable sets of gene families, Prokka (Seemann, 2014) (with default parameters) was employed for annotation of each genome. Get_Homologues (Contreras-Moreira and Vinuesa, 2013) was used for multiple local BLAST-directed comparisons among all genes (of all genomes), and these were further clustered by the OMCL method which drives the OrthoMCL algorithm (Li et al., 2003) within Get_Homologues. Subsequently compare_clusters.pl (a script within the same software) was used for retrieval of the set of orthologous genes uniquely present in all genomes (hereafter denominated the unicopy set). Mafft (Katoh and Standley, 2013) was used for multiple alignment of each unicopy gene, and then concatenation of all genes was done using FASconCAT (Kück and Meusemann, 2010). IQTree (Nguyen et al., 2015) was used for maximum likelihood (ML) estimation, with model choice employed before tree search, and branch support computed by UFBoot (Hoang et al., 2017).

### Effector Analysis

The aminoacid sequences of 62 effectors were retrieved from the Xanthomonas.org site (AvrBs2, AvrXccA1, AvrXccA2, HpaA, HrpW, XopA, XopAA, XopAB, XopAC, XopAD, XopAE, XopAF1, XopAF2, XopAG, XopAH, XopAI, XopAJ, XopAK, XopAL1, XopAL2, XopAM, XopAP, XopAU, XopAV, XopAW, XopAX, XopAY, XopAZ, XopB, XopC1, XopC2, XopD, XopE1, XopE2, XopE3, XopF1, XopF2, XopG1, XopG2, XopH1, XopI1, XopJ1, XopJ2, XopJ3, XopJ4, XopJ5, XopK, XopL, XopM, XopN, XopO, XopP, XopQ, XopR, XopS, XopT, XopU, XopV, XopW, XopX, XopY, and XopZ1) (Supplementary Table S1), to infer their evolution across the 31 genomes. For each genome, we assessed whether each effector without a premature stop codon had a frameshift or not. In order to do so, we performed local tBLASTn searches within the Blast + suite (Camacho et al., 2009) with an *e*-value of 1e-50 (a threshold obtained by trial-and-error, that minimized the number of extra hits bearing indels and mismatches without compromising detection of supposedly functional copies), discarding alignments in which the subject sequences aligned to less than 60% of the query length or with less than 60% identity. After tBLASTn runs, multiple alignments were generated by an in-house python script for each effector, each of which was manually checked in Aliview (Larsson, 2014) for detection of frameshifts and premature stop codons. Optimization of character evolution for each effector along the ML tree (i.e., presence, frameshifts without premature stop codons, sequences with premature stop codons, and absence) was obtained by the ace function within the R library phytools (Revell, 2012).

### Additional Gene Analyses

Additional gene families were investigated based on OrthoMCL clustering (Li et al., 2003) and STRING (Snel et al., 2000). OrthoMCL was run with default parameters, and results were then processed in the OrthologSorter pipeline (Setubal et al., 2018). Additionally, we created an Ortholog Alignment using gene families provided by OrthoMCL, with the A306 strain as anchor and all the XauB and XauC genomes, plus *X*. *citri* pv. *fuscans* 4384. This alignment is useful to visualize syntenic regions among genomes. The parts of this alignment that were used in reporting results in this work are shown in a simplified version in Supplementary Table S2. In the case of STRING, for each family of interest, the relevant genes as present in A306 were used as queries.

### Gum Production Assay

The xanthan gum production assays were performed as described by Moreira et al. (2010), without modification.

### Biofilm Production Assay

Biofilm production assays were performed following O’Toole (O’Toole, 2011), with a few modifications. The bacterial isolates were grown in liquid LB or XVM2 medium at 28°C. Bacterial density was standardized for all the isolates in OD600nm equal to 1.0. The samples were diluted 1:10 in liquid LB and 100 μL of each sample were placed in the 96-well plate for growth during 96 h at 28°C. After the incubation period, the plate was washed with distilled water to remove the cells and left drying for 2 h. Subsequently, 125 μL of crystal violet solution 0.1% (CV) were transferred to each well, which were left resting for 45 min. After the incubation period the plate was washed again with distilled water and left drying once more. Next, 125 μL of 95% ethanol were added to each well, which were left to rest for 45 min to complete CV dissolution. The absorbance reading was done at OD550nm. For each bacterial isolate 6 replicates were performed.

### Autoaggregation Assay

The autoaggregation assay was adapted from Alamuri et al. (2010), with modifications. Cultures of different bacterial isolates were grown at 28°C in liquid LB medium or XVM2: 1.16 g/L NaCl, 1.32 g/L (NH_4_)2SO_4_, 0.021 g/L KH_2_PO_4_, 0.055 g/L K_2_HPO_4_, 0.0027 g/L FeSO_4__⋅_7H_2_O, 1.8 g/L fructose, 3.423 g/L sucrose, 5 mMMgSO_4_, 1 mM CaCl_2_, 0.03% Casamino acid (pH 6.7), in triplicate. Samples with 10 mL of each culture were placed in a sterile 20 mL tube. Initially all cultures were vigorously shaken for 15 s and the tubes remained static throughout the experiment. Aliquots containing 100 μL were removed from approximately 1 cm of the top of the culture of each tube over time and optical density was measured at OD600 nm every hour_._

## Results

Information about the genomes that were sequenced for this work is given in Table 2. The additional genomes listed there were included in the analysis of pathogenicity-related genes.

**TABLE 2:** List of genomes used in the comparative analysis section, including information about the seven newly sequenced XauB and XauC genomes.

**Strain**	**Short name**	**Source**	**Replicons**	**Status**	**Length (bp)**	**CDS**	**Accession**
*X. citri* pv. a*urantifolii* FDC 535	XauC 535	*This work*	Chromosome	372 contigs – N50 37-kb	5,226,212	4,364	LAUH00000000.1
*X. citri* pv. a*urantifolii* FDC 763	XauC 763	*This work*	Chromosome	314 contigs – N50 35-kb	4,984,643	4,045	LAUI00000000.1
*X. citri* pv. a*urantifolii* FDC 867	XauC 867	*This work*	Chromosome	303 contigs – N50 41-kb	5,014,602	4,085	LAUJ00000000.1
*X. citri* pv. a*urantifolii* FDC 1559	XauC 1559	*This work*	Chromosome pXfc38 pXfc43	complete	5,191,653 37,980 43,013	4,225 40 36	CP011160.1 CP011162.1 CP011161.1
*X. citri* pv. a*urantifolii* FDC 1609	XauC 1609	*This work*	Chromosome pXfc32 pXfc37 pXfc46	complete	5,164,518 32,021 37,089 46,049	4,175 34 42 38	CP011163.1 CP011165.1 CP011164.1 CP011166.1
*X. citri* pv. a*urantifolii* ICPB 10535	XauC 10535	NCBI	Chromosome	352 contigs – N50 29-kb	5,012,633	4,034	ACPY00000000.1
*X. citri* pv. a*urantifolii* ICPB 11122	XauB 11122	NCBI	Chromosome	244 contigs – N50 38-kb	4,879,662	3,918	ACPX00000000.1
*X. citri* pv. a*urantifolii* FDC 1561	XauB 1561	*This work*	Chromosome pXfb33	complete	4,993,063 33,022	4,048 42	CP011250.1 CP011251.1
*X. citri* pv. a*urantifolii* FDC 1566	XauB 1566	*This work*	Chromosome pXfb35	complete	4,926,567 35,521	3,983 44	CP012002.1 CP012003.1
*X. citri* pv. *fuscans* str. 4834-R	Xfus 4834	NCBI	Chromosome pla plb plc	complete	4,981,995 45,224 19,514 41,950	3,977 51 22 48	FO681494.1 FO681495.1 FO681496.1 FO681497.1
*X. citri* pv. *citri* str. 306	Xac 306 or A306	NCBI	Chromosome pXAC33 pXAC64	complete	5,175,554 33,700 64,920	4,321 42 73	AE008923.1 AE008924.1 AE008925.1

### Phylogenomic Analyses

**FIGURE 1:**
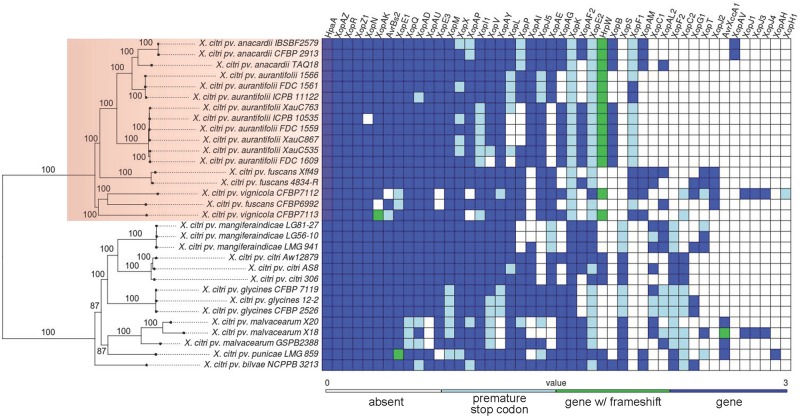
Maximum likelihood phylogeny of 31 *Xanthomonas citri* strains and table of presence, absence, and pseudogene status of 46 effector genes.

For the phylogenomic analyses we used 31 genomes (Table 3). Gene family computation resulted in 2,449 single-copy shared families, leading to a concatenated alignment of 2,516,841 bp. The best ML model was GTR + G + R2 (where R2 means a mixed model of rate variation with two rate classes), with most nodes with support ≥ 95%. The resulting phylogeny is shown in Figure 1.

**TABLE 3:** List of 31 genomes used in the phylogenomic analysis.

**Strain name**	**NCBI name**	**NCBI assembly**
*X. citri* pv. anacardii CFBP 2913	*X*. *citri* pv. *anacardii* CFBP 2913	GCF_002688625.1
*X. citri* pv. *anacardii* IBSBF2579	*X*. *citri* pv. *anacardii* IBSBF2579	GCF_002837255.1
*X. citri* pv. *anacardii* TAQ18	*X*. *citri* pv. *anacardii* TAQ18	GCF_002898415.1
*X. citri* pv. *aurantifolii* ICPB 11122	*X*. *fuscans* subsp. *aurantifolii* ICPB 11122	GCF_000175135.1
*X. citri* pv. *aurantifolii* FDC 1561	*X*. *fuscans* subsp. *aurantifolii* FDC 1561	GCF_002079965.1
*X. citri* pv. *aurantifolii* 1566	*X*. *fuscans* subsp. *aurantifolii* 1566	GCF_001610915.1
*X*. *citri* pv. *aurantifolii* ICPB 10535	*X*. *fuscans* subsp. *aurantifolii* ICPB 10535	GCF_000175155.1
*X. citri* pv. *aurantifolii* FDC 1559	*X*. *fuscans* subsp. *aurantifolii* FDC 1559	GCF_001610795.1
*X*. *citri* pv. *aurantifolii* FDC 1609	*X*. *fuscans* subsp. *aurantifolii* FDC 1609	GCF_001610815.1
*X*. *citri* pv. *aurantifolii* XauC535	*X*. *citri* pv. *aurantifolii* XauC535	GCF_004329265.1
*X*. *citri* pv. *aurantifolii* XauC763	*X*. *citri* pv. *aurantifolii* XauC763	GCF_004329275.1
*X*. *citri* pv. *aurantifolii* XauC867	*X*. *citri* pv. *aurantifolii* XauC867	GCF_004329295.1
*X*. *citri* pv. *bilvae* NCPPB 3213	*X*. *citri* pv. *bilvae* NCPPB 3213	GCF_001497855.1
*X*. *citri* pv. *citri* 306	*X*. *axonopodis* pv. *citri* 306	GCF_000007165.1
*X*. *citri* pv. *citri* AS8	*X*. *citri* pv. *citri* AS8	GCF_000950875.1
*X*. *citri* pv. *citri* Aw12879	*X*. *citri* subsp. *citri* Aw12879	GCF_000349225.1
*X*. *citri* pv. *fuscans* 4834-R	*X*. *fuscans* subsp. *fuscans* 4834-R	GCF_000969685.2
*X*. *citri* pv. *fuscans* CFBP6992	*X*. *citri* pv. *phaseoli* var. *fuscans* CFBP6992	GCF_002759335.1
*X*. *citri* pv. *fuscans* Xff49	*X*. *citri* pv. *fuscans* Xff49	GCF_002309515.1
*X*. *citri* pv. *glycines* 12-2	*X*. *citri* pv. *glycines* 12-2	GCF_002163775.1
*X. citri* pv. *glycines* CFBP 2526	*X*. *axonopodis* pv. *glycines* CFBP 2526	GCF_000495275.1
*X*. *citri* pv. *glycines* CFBP 7119	*X*. *axonopodis* pv. *glycines* CFBP 7119	GCF_000488895.1
*X*. *citri* pv. *malvacearum* GSPB2388	*X*. *citri* pv. *malvacearum* GSPB2388	GCF_000309925.1
*X*. *citri* pv. *malvacearum* X18	*X*. *citri* pv. *malvacearum* X18	GCF_000454505.1
*X*. *citri* pv. *malvacearum* X20	*X*. *citri* pv. *malvacearum* X20	GCF_000454525.1
*X*. *citri* pv. *mangiferaindicae* LG56-10	*X*. *citri* pv. *mangiferaeindicae* LG56-10	GCF_002920975.1
*X*. *citri* pv. *mangiferaindicae* LG81-27	*X*. *citri* pv. *mangiferaeindicae* LG81-27	GCF_002926255.1
*X*. *citri* pv. *mangiferaindicae* LMG 941	*X*. *citri* pv. *mangiferaeindicae* LMG 941	GCF_000263335.1
*X*. *citri* pv. *punicae* LMG 859	*X*. *axonopodis* pv. *punicae* LMG 859	GCF_000285775.1
*X*. *citri* pv. *vignicola* CFBP7112	*X*. *citri* pv. *vignicola* CFBP7112	GCA_002218265.1
*X*. *citri* pv. *vignicola* CFBP7113	*X*. *citri* pv. *vignicola* CFBP7113	GCF_002218285.1

### Xanthan Gum, Biofilm, and Autoaggregation Analysis

In an attempt to understand which physiological factors could contribute to the induction of the respective virulence phenotypes of the investigated strains, xanthan gum production, biofilm, and cell self-aggregation were analyzed (Supplementary Table S3). As expected, A306 is the strain with the highest production of xanthan gum by bacterial mass. On the other hand, XauC 535 and XauC 1609 showed respectively the highest biofilm production and self-aggregation capacity in virulence-inducing medium (XVM2).

### Type III Secretion System Effector Analysis

Out of the 62 effectors investigated, four were present in all genomes, 16 were absent from all genomes, for a total of 42 effectors with variable presence/absence across lineages (Figure 1). For 11 effectors we observed interesting patterns of presence, absence, or pseudogenization. For these effectors we inferred their evolutionary history in terms of gains, losses or pseudogenization (Supplementary Figure S1).

### Other Pathogenicity-Related Genes

Individual genes or genes that collectively encode proteins that compose cell complexes involved in virulence and adaptation were analyzed in all genomes listed in Table 2. The virulence and adaptation genes were grouped into two broad categories: (1) secretion systems (other than Type III effectors); (2) surface structure. The analysis framework we have adopted is as follows. The A306 strain has several genes in each of the categories analyzed (da Silva et al., 2002). On the other hand, as will be seen, the XauB and XauC genomes that we have analyzed lack many or all of the genes in some of these categories. In order to better understand the potential impact that the lack of these genes may have in the pathogenicity and/or survival capabilities of the XauB and XauC strains, for each category in which XauB and XauC lack genes we first describe the A306 gene content. We then note the differences exhibited by XauB and XauC (as given by the Ortholog Alignment of 11 genomes, with A306 as anchor, as described in section Materials and Methods), followed by a network analysis based on the A306 genes, using the tool STRING (Snel et al., 2000).

### Secretion Systems

We verified that all the analyzed genomes retain all orthologous genes belonging to the two gene clusters associated with synthesis of the type II secretion system (T2SS, XAC0694-XAC0705, and XAC3534-3544), all the genes involved in structuring the type III secretion system apparatus (T3SS, XAC0393-XAC0417), all the genes associated with the type VI secretion system (T6SS, XAC4119-20-24, XAC4139-40-45), as well as complete Sec and Tat secretion systems. The main differences observed are related with the type I secretion system (T1SS), the type IV secretion system (T4SS) and effectors of the type III secretory system (T3SS). Results for T3SS effectors were already presented above.

### XauB and XauC Lack Key Genes in the Type I Secretion System

The T1SS corresponds to an ABC transporter system and it is basically composed of two proteins, HlyD – an ABC transporter, and HlyB – a membrane fusion protein, whose main function, together with TolC, is to promote the secretion of toxins (Koronakis et al., 2004). In A306 two copies of the gene encoding the toxin presumably secreted by this system, hemolysin (type-calcium, XAC2197-98), are upstream of the genes *hlyDB* (XAC2201-02), separated by two hypothetical proteins (XAC2199-2200). These gene families (XAC2197-2202) were not found in the XauB genomes. The XauC genomes on the other hand do not have orthologs of XAC2197-98, but they do have *hlyB* and *hlyD*. Other genes associated with synthesis and regulation of hemolysin in these genomes were also analyzed. All genomes have orthologs of XAC4303 and XAC1668 (cryptic hemolysin transcriptional regulator), XAC3043 and XAC0079 (hemolysin III, *hly*3), and XAC1709 (hemolysin, *tlyC*). However, in XauB and XauC strains we did not find orthologs for the genes XAC1814 (outer membrane hemolysin activator protein) and XAC1918 (hemolysin-like protein).

**FIGURE 2:**
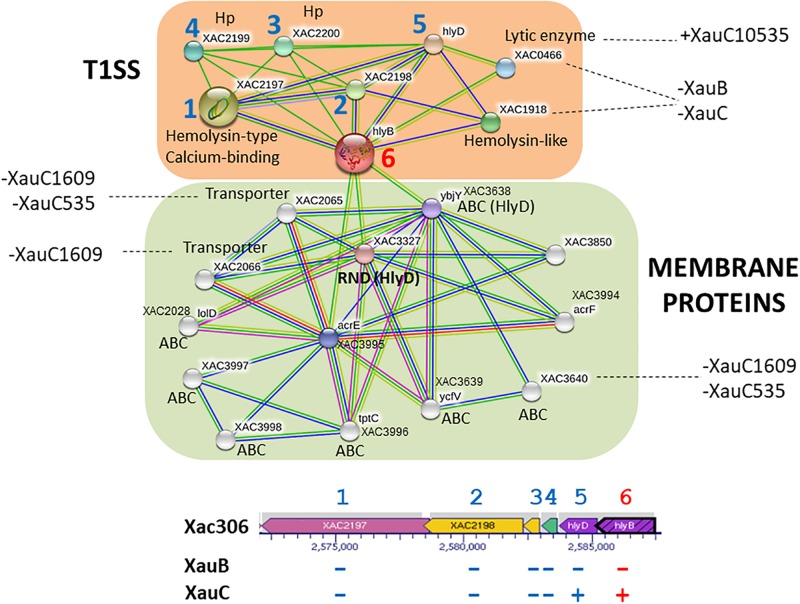
Network of putative interactions between genes/proteins using as query the *hlyB* gene from strain A306, a key gene in the Type 1 Secretion System. The nodes represent the genes/proteins present in the A306 genome. The color codes on the lines is: red – fusion evidence; green – neighborhood evidence; blue – co-occurrence evidence; purple – experimental evidence; yellow – text mining evidence; light blue – database evidence; black – co-expression evidence. The network part of the figure was generated by the program STRING (Snel et al., 2000). Genomic context is shown at the bottom, based on a figure generated by the BioCyc resource (Karp et al., 2017). The arrows represent the genes and the gray background the transcriptional units. The blue numbers correlate the position of a given gene in the genomic context and the same gene on the network. The number in red has a similar purpose and simply highlights the query gene in the network inference.

Analysis of possible interactions of the products of genes *hly*B and *hly*D (Figure 2) revealed two well-defined interaction networks for the A306 *hly*B gene used as query to STRING. One of these groups, in orange background, is the genes/proteins associated with T1SS composition and functionality. The other network (green background) is composed primarily of membrane genes/proteins, essentially ABC transporters. Eight genes/proteins represented by the nodes of the network composing the T1SS apparatus correspond to the same genes in the cluster discussed above, including the gene encoding the lytic enzyme (XAC0466) present in the XauC10535 genome. We observed that the genomes of XauB strains do not have any of these genes. However, the loss of a single gene of hemolysin in XauC strains would have a small effect, since this loss could be compensated by paralogous genes in their genomes. Concerning the cluster of membrane proteins, three of the ABC transporters are associated with resistance to acriflavin (XAC3994-95 and XAC3850) and two have the hlyD domain (PF00529), involved with secretion of toxins. Some of the genes in this network were not found in the genomes of strains XauC 1609 and XauC 535.

### XauB and XauC Genomes Lack the Chromosomal Copy of Type IV Secretion System Genes

The genes encoding the T4SS in A306 are found in two similar gene clusters, one in the chromosome (XAC2607-2623) and another in the plasmid (XACb0036-b0047) (da Silva et al., 2002). The genomes of XauB and XauC have only the plasmidial cluster (Supplementary Table S2). Note that in the cases of XauB 11122 and XauC 10535, whose contigs are not distinguished as belonging to the chromosome or to a plasmid, it is our inference based on synteny that the T4SS genes actually belong to a plasmid (Supplementary Table S2).

### XauB and XauC Genomes Lack Key Genes in the Synthesis and Regulation of Type IV Pilus

A306 has at least four clusters of genes involved with synthesis and regulation of type IV pilus (T4p). One of these clusters, *pilE-Y1-X-W-V-fimT* (XAC2664-2669) is found between a set of prophage genes upstream and a transposase downstream, suggesting possible horizontal gene transfer. We observed that two of these genes (*pilX-pilV*) are missing in the XauB and XauC genomes (Supplementary Table S2). In the case of cluster *pilS-R-B-A-A-C-D* (XAC3237-3243) (Yang et al., 2004), the XauB and XauC genomes lack the two copies of *pilA. PilA* encodes pilin, an essential T4p component that contributes to twitching motility and biofilm development in A306 (Dunger et al., 2014; Petrocelli et al., 2016). Another gene whose product has a function related to T4p is *pilL* (XAC2253). In A306 this gene is found in a large genomic island (XAC2176 to XAC2286), but is absent from the XauB and XauC genomes.

**FIGURE 3:**
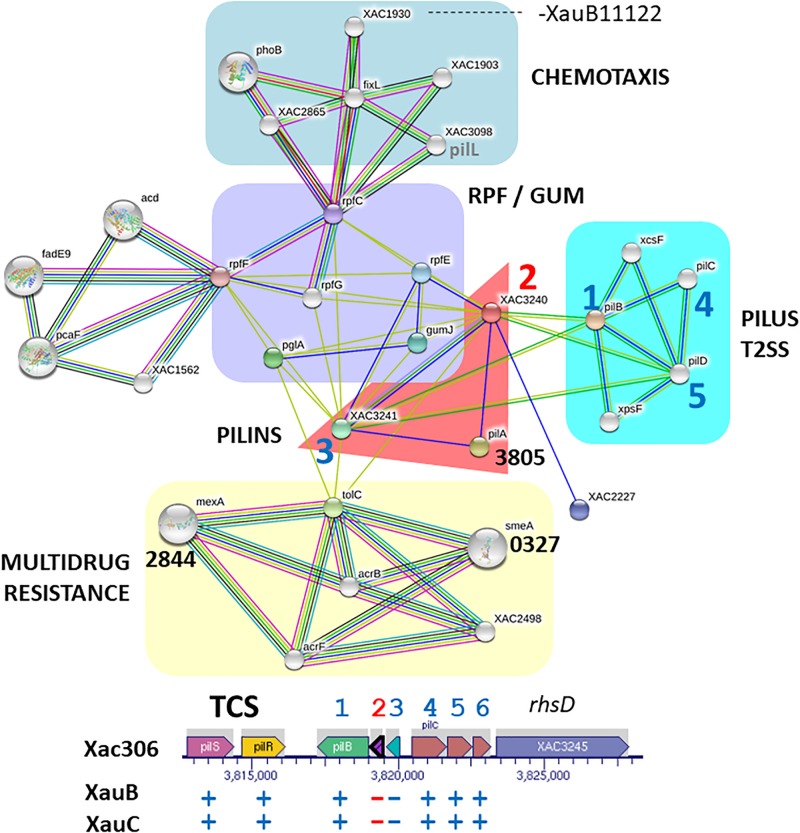
Network of putative interactions between genes/proteins using as query the pilin gene XAC3240 from the A306 strain. For additional details see legend of Figure 2. The network figure was generated by the program STRING (Snel et al., 2000) and the genomic context figure was based on a figure generated by the BioCyc resource (Karp et al., 2017).

We carried out an analysis of predicted interactions of *pilA* (XAC3240) (Figure 3). In orange background we observe that XAC3240 interconnects five other networks and that the pilin subunits (XAC3240 and XAC3241) are connected to one another, and connect to another *pilA* (XAC3805). As expected, one of the networks starting from pilins refers to genes/proteins associated with the pilus structure and with the T2SS (cyan background), as is known that both are evolutionarily related (Peabody et al., 2003). Moreover, three genes share the same genomic region of pilins in the chromosome of A306 (1, 4, and 5). Close to the pilin network (purple background) there is a network involving genes associated with quorum sensing (*rpf*), gum synthesis (*gum*) and the plant tissue degrading enzyme polygalacturonase (*pglA*), known to be virulence-related (Wang et al., 2008). Likewise, this network reflects the interaction profiles of DSF production mediated by *rpf* genes, which act as signaling molecules of gum synthesis and consequently of the production of plant cell wall degrading enzymes, as is the case for PglA mediated by T2SS (Vojnov et al., 2001; An et al., 2013). In addition, another network expands from *rpfC*. Indeed, this network (pale green background), associated with chemotaxis-related genes, includes *phoB*, which is involved in phosphate regulons, essential for adaptation and virulence induction in members of the genus *Xanthomonas* (Pegos et al., 2014; Moreira et al., 2015).

### XauB and XauC Genomes Lack an Alginate Biosynthesis Gene

In A306, the first gene downstream of *pilE-Y1-X-W-V-FimT*, XAC2670, encodes an alginate biosynthesis protein, which is absent from the XauB and XauC genomes (Supplementary Table S2). The A306, XauB, and XauC genomes encode another gene whose product is involved with the metabolism of alginate, alginate lyase: *algL* (XAC4349).

**FIGURE 4:**
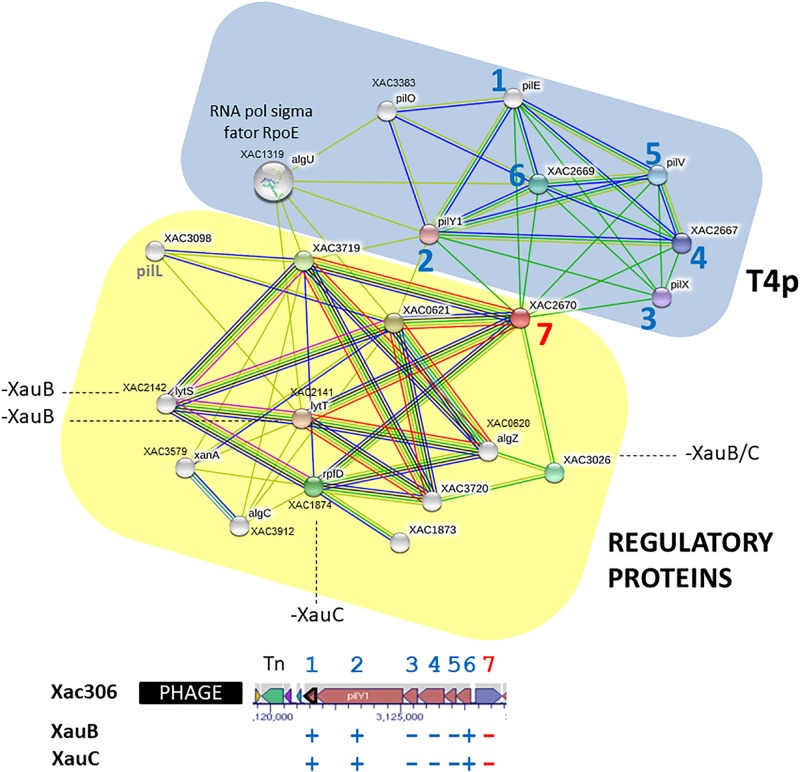
Network of putative interactions between genes/proteins using as query the A306 gene XAC2670, which encodes an alginate biosynthesis protein. For additional details see legend of Figure 2. The network figure was generated by the program STRING (Snel et al., 2000) and the genomic context figure was based on a figure generated by the BioCyc resource (Karp et al., 2017).

Analysis of predicted interactions of the gene XAC2670 with other genes/proteins revealed two distinct clusters (Figure 4). The first one on blue background contains seven nodes whose genes/proteins are directly related to synthesis and regulation of T4p (*pil* genes previously described). In this group, excepting *pilO* (XAC3383), all other genes are present in a cluster (XAC2664-XAC2669) downstream of a transposase and a phage insertion (numbers 1–6), and upstream of the gene XAC2670. The second cluster, on yellow background, contains 12 nodes, with most genes/proteins related to regulatory functions, especially *algZR* (XAC0620-21), encoding a two-component system, respectively for sensor and regulatory proteins (Okkotsu et al., 2014), and *algC* (phosphomannomutase) (Davies and Geesey, 1995), all described as essential to alginate synthesis. Another two-component system, *lytST* (XAC2142-2141, sensor-regulator), is also present in this network. *LytT*, as well as *rpfD*, also present in the yellow background network (XAC1874) and member of the *rpf* gene cluster, exhibit the lytR domain, also present in proteins such as AlgR with DNA binding function (Nikolskaya and Galperin, 2002). Finally, *rpoE* (XAC1319) connects the two clusters (Figure 4), and therefore may be directly associated with both by regulating the EPS synthesis and/or by modulating T4p expression.

### XauB and XauC Lack Several Genes Related to Hemagglutinin and Hemolysin Synthesis

A further set of genes with significant differences in terms of presence and absence in the analyzed genomes is related to hemagglutinin and hemolysin synthesis. These genes are located in two regions in the genome of A306 (XAC4112-XAC4125 and XAC1810-XAC1819). The first region is flanked by genes that are part of the T6SS, both downstream and upstream. All genes of this region are present in all genomes of XauB and XauC. However, the genes in the second region (XAC1810-XAC1819) are totally absent in the XauB and XauC genomes. Among these genes we highlight *fhaC* (XAC1814), which codes for an outer membrane hemolysin activator, *fhaB* (XAC1815), which codes for a filamentous hemagglutinin, XAC1816, which codes for a hemagglutinin/hemolysin-related protein, XAC1818, which codes for hemagglutinin, and the genes in the operon *HmsHFR-hp* (XAC1813-1810).

**FIGURE 5:**
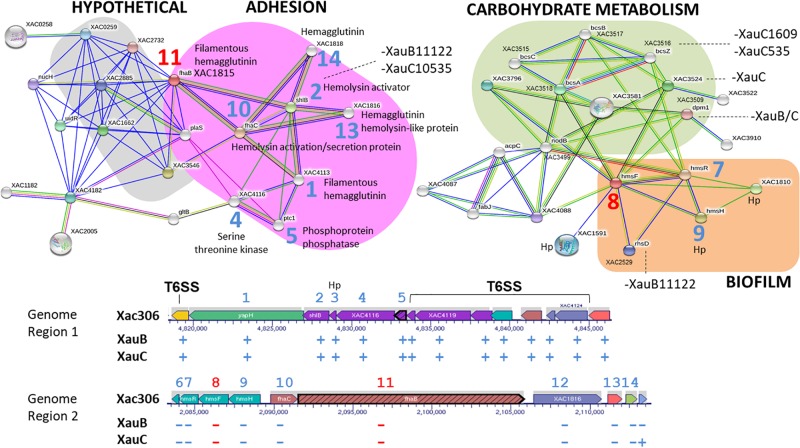
Networks of putative interactions between genes/proteins using as queries the A306 genes *fhaB* (XAC1815) and *hmsF* (XAC1812). For additional details see legend of Figure 2. The network figure was generated by the program STRING (Snel et al., 2000) and the genomic context figure was based on a figure generated by the BioCyc resource (Karp et al., 2017).

Analysis of predicted interactions of the gene *fhaB* (XAC1815) allowed the characterization of two major interaction networks (Figure 5). One of these networks (pink background) is associated with adhesion, whereas the other network (gray background) basically contains hypothetical genes/proteins. Furthermore, other genes/proteins in the adhesion network are located in the two regions related to hemagglutinin and hemolysin synthesis mentioned in the previous paragraph.

Using *hmsF* (XAC1812), we obtained an interaction network made of three clusters, two of which seem to be functionally related (Figure 5). One of the clusters (green background) contains genes/proteins associated with carbohydrate metabolism. The other cluster (orange background) contains the *hmsFHR* genes, related to biofilm formation.

**FIGURE 6:**
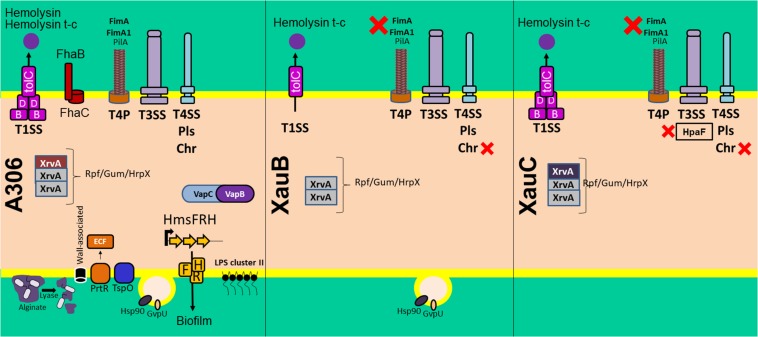
Summary of the comparative analyses of 11 *Xanthomonas citri* genomes listed in Table 2 in terms of the pathogenicity-related genes and systems discussed in the text. The red X symbol denotes lack of the genes or system next to it.

A summary of these results is presented in Figure 6, which includes some additional pathogenicity-related genes also lacking in the XauB and XauC genomes: *vapBC*, a toxin-antitoxin module in *Acidovorax citrulli* (Shavit et al., 2015); and *tspO*, which encodes a protein with a potential role in the oxidative stress response, iron homeostasis, and virulence expression in *Pseudomonas* (Leneveu-Jenvrin et al., 2014).

## Discussion

Our results show that the three lineages inflicting citrus canker (A strains and XauB and XauC strains) can be robustly separated into two well defined clades, with A strains in one clade, which we call the Citri-citri (C-c) clade, and XauB and XauC in another clade, which we call the aurantifolii clade (Figure 1); furthermore, XauB and XauC were shown to be in a paraphyletic clade, with *X. citri* pv. *anacardii* being closer to XauB (Figure 1). It is noteworthy to observe that the C-c and aurantifolii clades contain strains that are pathogenic in taxonomically disparate plant hosts, such as citrus (*X. citri* pv. citri and *X. citri* pv. *aurantifolii*), leguminosae (*X. citri* pv. *glycines* and *X. citri* pv. *fuscans* emerging from more basal nodes), cashew (*X. citri* pv. *anacardii*), mango (*X. citri* pv. *mangiferaindicae*), and cotton (*X. citri* pv. *glycines*). Curiously, *X. citri* pv. *anacardii* (infecting cashew) apparently evolved within a citrus-associated clade, suggesting a possible host jump.

We have made an extensive analysis of the presence and absence of effectors in the 31 genomes we sampled to reconstruct the phylogeny. We now discuss the main results of this analysis. *XopS* was shown to be the only effector among the 62 investigated that is present in the C-c clade (although in some cases as a pseudogene) but absent in the aurantifolii clade (Figure 1). XopS is completely dependent on HpaB to be translocated; it contributes to disease symptoms and bacterial growth and suppresses pathogen-associated molecular pattern (PAMP)-triggered plant defense gene expression (Schulze et al., 2012). *XopF1* was found to have the opposite pattern compared to *xopS*: present in the aurantifolii clade (in some cases as a pseudogene) and absent in the C-c clade. In *Xanthomonas oryzae* pv. *oryzae*, XopF1 has been shown to repress basal PAMP-triggered immunity response in rice (Mondal et al., 2015). A third interesting case is *xopK*, which is present in the C-c clade, but was found to be a pseudogene in all genomes of the aurantifolii clade. XopK has been shown to inhibit PAMP-triggered immunity upstream of mitogen-activated protein kinase cascades in *Xanthomonas oryzae* pv. *oryzae* (Qin et al., 2018). Figure 1 makes clear that there are many other differences in effector repertoires among the 31 genomes analyzed; 11 of these other effectors have been studied in terms of their gains and losses across the evolution of the 31 strains (Supplementary Figure S1). Because the pattern of gains, losses and pseudogenization is more intricate, additional studies are required to correlate these inferred histories to known phenotypic traits of the affected strains.

In addition to effectors, we have carefully analyzed the gene content in the broad categories of secretion systems-related genes and surface structure-related genes. Our main tool in this analysis, in addition to presence/absence results, was the prediction of possible interactions. These analyses resulted in several noteworthy differences of XauB and XauC strains when compared to the A306 genome.

### Type I Secretion System

The T1SS is responsible for secreting toxins, such as hemolysins in *E. coli* (Thomas et al., 2014). In the three XauB strains investigated, both genes coding for apparatus secretory proteins (*hlyB* and *hlyD*) and genes coding for hemolysins (*hlyA*) were not found. This absence might contribute to a decrease in the elicitation of the plant immune response as well as to decreased competitive capability with other organisms due to the inability to secrete these toxins.

### Type IV Secretion System

The T4SS has multiple functions, including transport of a variety of substrates from DNA and protein-DNA complexes to proteins, and it plays fundamental roles in both bacterial pathogenesis and adaptation to the cellular milieu in which bacteria live (Darbari and Waksman, 2015). Jacob et al. (Jacob et al., 2014), reported that the T4SS in A306, unlike the T3SS, is not associated with virulence induction, but rather in cell-cell interactions. This finding was confirmed by Souza et al. (2015), who demonstrated the involvement of the chromosomal T4SS in bacterial killing, showing that this special class of T4SS is a mediator of both antagonistic and cooperative interbacterial interactions. We speculate that the lack of the T4SS chromosomal gene cluster in XauB and XauC genomes may have a consequence in the ability of these strains to compete with other bacteria, in particular with A306 itself. If this speculation is correct, this may be an explanation for the apparent disappearance of XauB strains from the field (Chiesa et al., 2013).

### Synthesis and Regulation of Type IV Pilus

Among the protein complexes involved in biofilm formation is the type IV pilus (T4p) (Dunger et al., 2014). Besides actively participating in this matrix, the T4p is of fundamental importance in the adhesion process to the host tissue in the early stages of infection and independent flagellum displacement, called twitching motility (Mattick, 2002). The orthologous gene cluster *pilE-Y1-X-W-V-FimT* (XAC2664-2669) of A306 in *P. aeruginosa* has been shown to be involved in negative regulation of swarming motility (Giltner et al., 2010; Kuchma et al., 2012). In this same context, inactivation of *pilA* inserted in the *pilS-R-B-A-A-C-D* cluster (XAC3238-3243) interfered with twitching motility, biofilm development, and adherence of XAC (Dunger et al., 2014). Thus, the lack of genes *pilX*, *pilV*, *pilA*, *pill*, and *fimT* genes in the XauB and XauC genomes, all involved with T4p apparatus structuring, seem to explain at least in part the decreased production of biofilm and self-aggregation capability in some XauB and XauC strains (Supplementary Table S3). On the other hand, these same results show that XauC 535 and XauC 1609 presented, respectively, the highest biofilm production and self-aggregation capability in virulence-inducing media (XVM2) among all strains, even in the absence of the genes listed; this result requires further investigation.

### Alginate Biosynthesis

Alginate is an EPS related to biofilm formation and produced by bacteria of the genus *Pseudomonas* (Baker and Svanborg-Eden, 1989; Orgad et al., 2011). The function of alginate lyase is to hydrolyze bonds that hold the structured polymer, thereby enabling the bacterium to leave the biofilm structure, allowing its spreading by the colonized tissue (Boyd and Chakrabarty, 1994).

The intricate network we inferred for XAC2670 (which codes for an alginate biosynthesis protein in A306) may be depleted in XauB and XauC due to the lack of key genes/proteins in the composition of these clusters, as it is the case of *pilX* and *pilV*, and XAC2670 itself, which could impair the synthesis and regulation of T4p apparatus and EPS production. Importantly, there are no reports in the literature mentioning any *Xanthomonas* species as an alginate producer. However, it is interesting to notice the presence of at least nine genes in A306 that may be involved with synthesis and regulation of this polymer, from which four are present in the interaction networks described above.

### Hemagglutinin and Hemolysin Synthesis

The hemagglutinin gene (XAC1818) has been described as fundamental to the virulence process in many organisms, including *Xylella fastidiosa* (Caserta et al., 2010; Voegel et al., 2010), another plant pathogen of the Xanthomonadaceae family, and in A306 (Gottig et al., 2009). The *hmsHFR-hp* genes (XAC1813-1810) are involved in adaptation and virulence, and have been reported respectively to be homologous to *E. coli* genes *pgaABCD* (Wang et al., 2004). Mutations in genes from this operon in members of the genera *Chromobacterium*, *Yersinia*, and *Xanthomonas* have resulted in reduction of biofilm formation and consequent reduction in virulence induction (Becker et al., 2009; Abu Khweek et al., 2010; Wang et al., 2012). Therefore, the absence of XAC1810-XAC1819 in the genomes of strains XauB and XauC might contribute to less efficient tissue adhesion processes and biofilm formation, and reduce cell-to-cell aggregation dependent of adhesin and exopolysaccharides molecules; this in turn would lead to reduction in tissue colonization capabilities in these strains.

## Conclusion

Taken together, our results show that the XauB and XauC genomes lack many genes that are known to play a role in host infection, either in A306 or in other pathosystems. This result is consistent with the attenuated citrus canker phenotypes of the XauB and XauC strains. In addition, the lack of recent reports about the presence of XauB and XauC strains in the field suggests a scenario in which A306 or other A strains may have outcompeted the XauB and XauC strains, possibly leading to their eradication. If so, this would be a process similar to what has taken place with *Candidatus Liberibacter americanus*, a causative agent of citrus huanglongbin, which has reportedly been eradicated, in South America, by *Candidatus Liberibacter asiaticus* (Wulff et al., 2014). It is to be hoped that such knowledge can be put to practical use in the efforts to eradicate from the field the A strains as well.

## Data Availability Statement

Raw reads are available at the Short Read Archive at NCBI at the following URLs: https://www.ncbi.nlm.nih.gov/Traces/study/?acc=PRJNA273983; https://www.ncbi.nlm.nih.gov/sra/?term=PRJNA273983.

## Author Contributions

JS, LM, JF, JB, and FJ conceived the study. JB and FJ selected and prepared strains for sequencing. AF, RF, and JF did the genome sequencing. AV and NA assembled the genomes. NF, ÉF, WC, AS, IC, CL, RA, CG, JM, JP, LM, and JS analyzed the data and interpreted the results. JS, LM, and JP wrote the manuscript.

## Conflict of Interest

The authors declare that the research was conducted in the absence of any commercial or financial relationships that could be construed as a potential conflict of interest.

## Footnotes

1 https://bitbucket.org/izhbannikov/seqyclean

2 https://www.pacb.com/documentation/smrt-analysis-software-installation-v2-3-0/

3 https://www.ncbi.nlm.nih.gov/projects/treeview/

## Supplementary Material

FIGURE S1 Trees with the reconstruction of gains, losses, and pseudogenization events for 11 effector genes. The effector name is shown at the top of each tree frame.

TABLE S1 Type III Secretion System Effectors investigated.

TABLE S2 Representation of an alignment of ortholog genes having as anchor the Xac306 genome.

TABLE S3 Results of biochemical assays related to xanthan gum and biofilm production, and aggregation (left-had side).
