# Supplementary figures and images for: Analyses of Seven New Genomes of Xanthomonas citri pv. aurantifolii Strains, Causative Agents of Citrus Canker B and C, Show a Reduced Repertoire of Pathogenicity-Related Genes
Source: Front Microbiol. 2019 Oct 11;10:2361. doi: 10.3389/fmicb.2019.02361 (PMC6797930; doi:10.3389/fmicb.2019.02361)

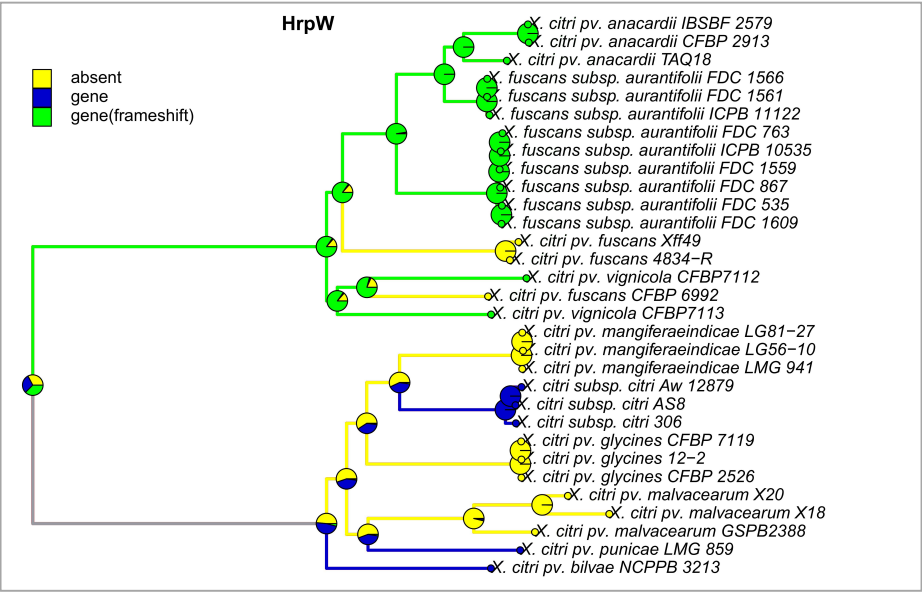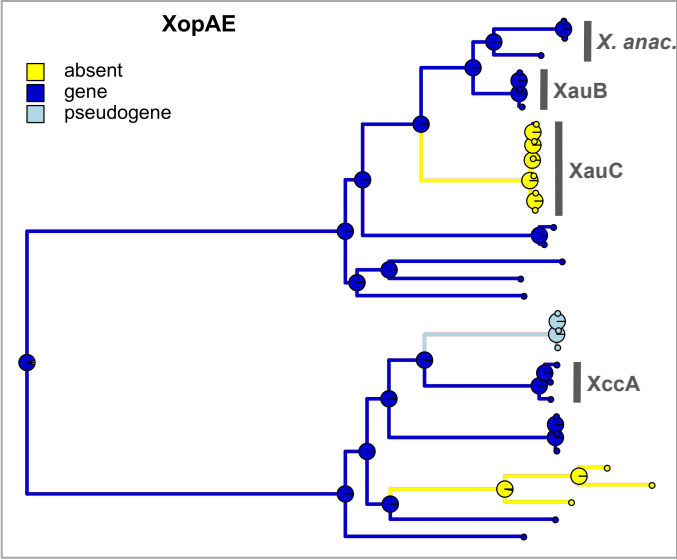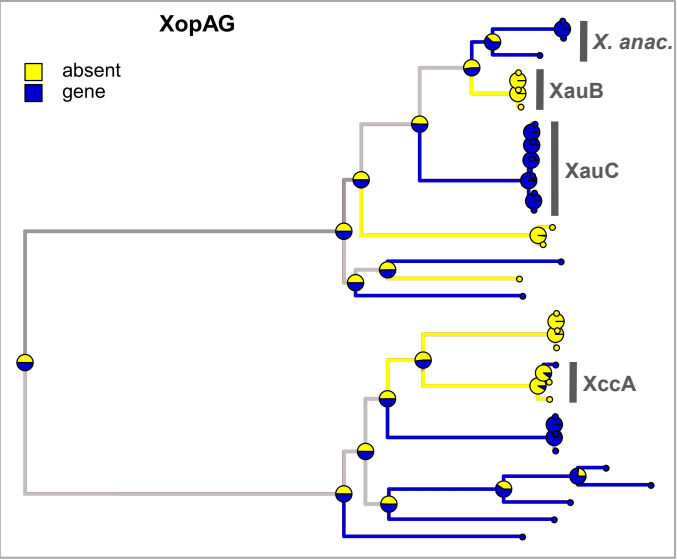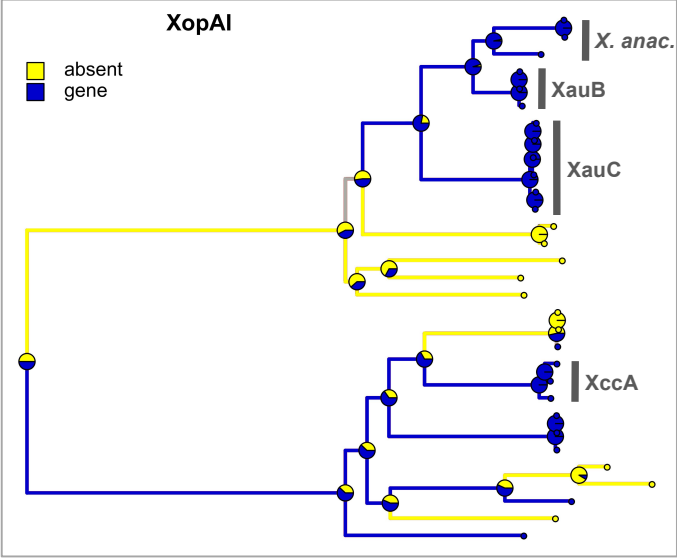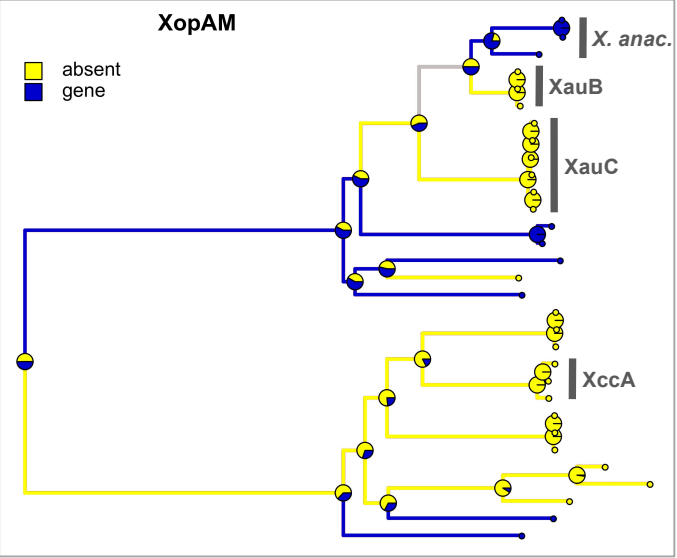

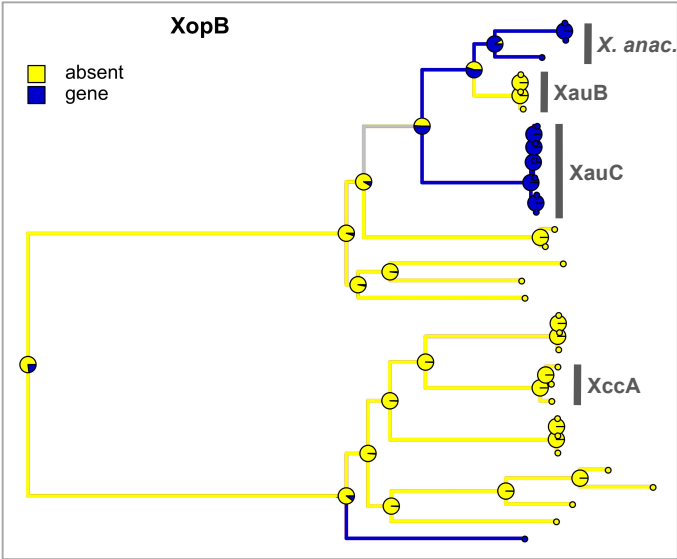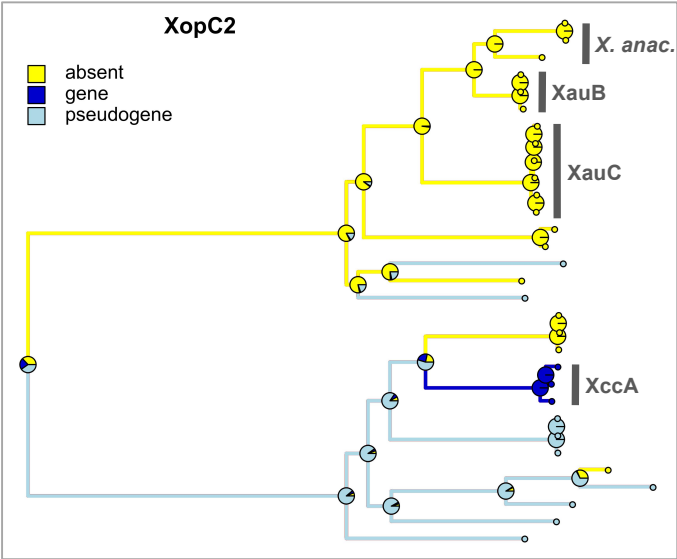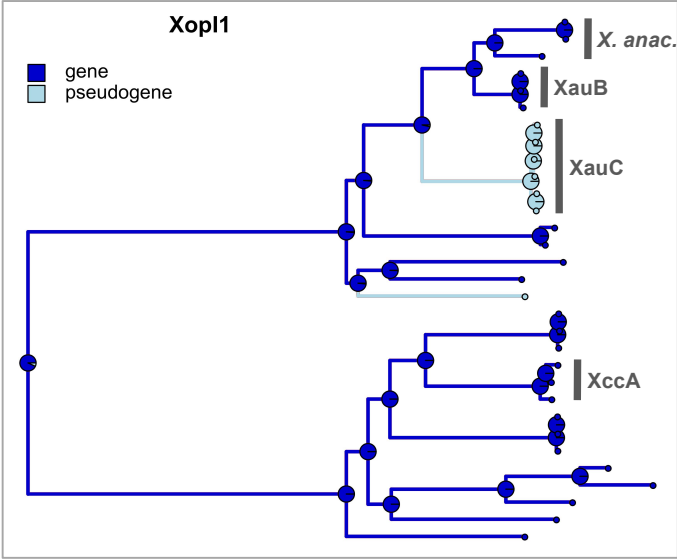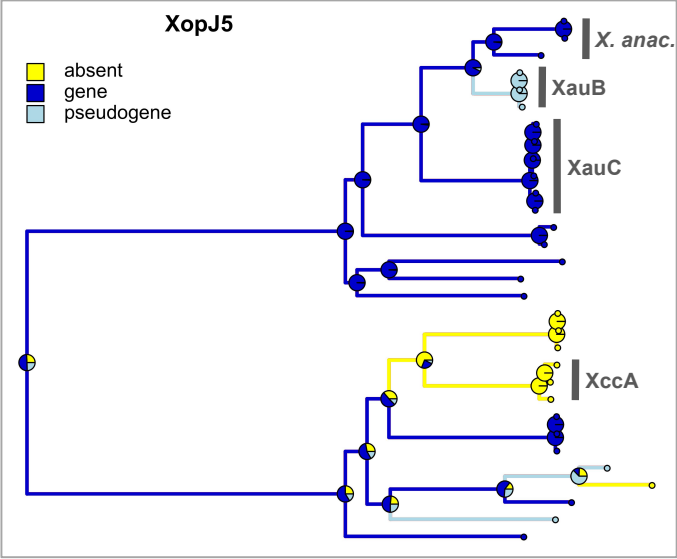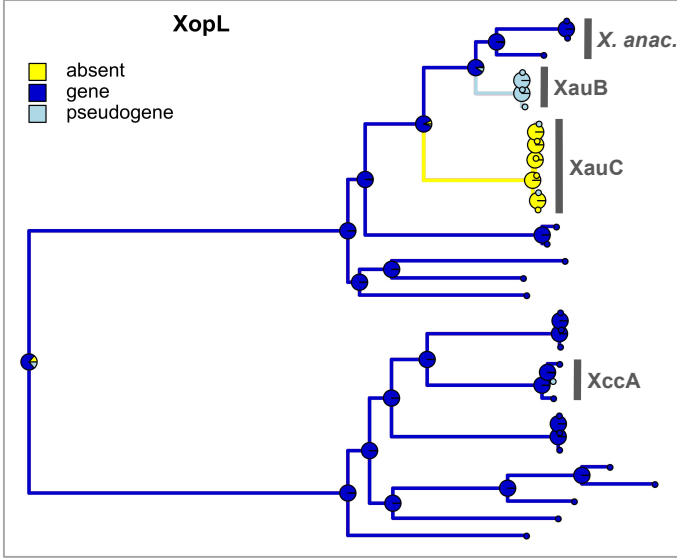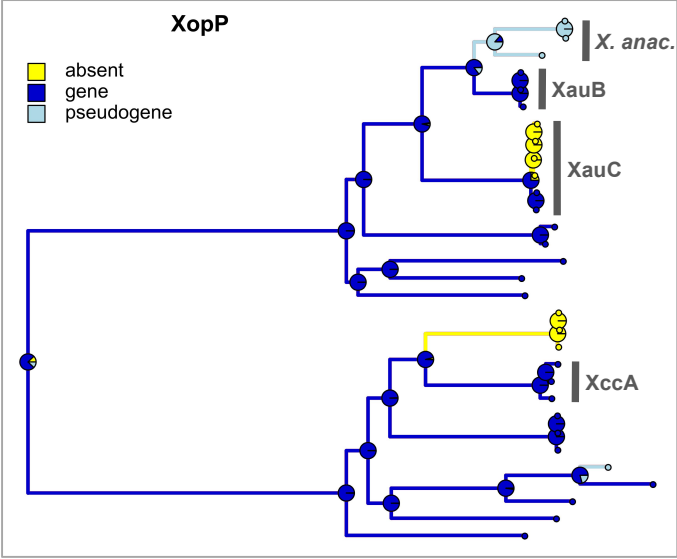

Supplement: FIGURE S1 — Trees with the reconstruction of gains, losses, and pseudogenization events for 11 effector genes. The effector name is shown at the top of each tree frame. [file Data_Sheet_1.PDF]
