# Supplementary material for: Analyses of Seven New Genomes of Xanthomonas citri pv. aurantifolii Strains, Causative Agents of Citrus Canker B and C, Show a Reduced Repertoire of Pathogenicity-Related Genes
Source: Front Microbiol. 2019 Oct 11;10:2361. doi: 10.3389/fmicb.2019.02361 (PMC6797930; doi:10.3389/fmicb.2019.02361)
Supplement: TABLE S1 — Type III Secretion System Effectors investigated. [file Table_1.DOCX]

**Table ST1:** Type III Secretion System Effectors investigated. The names follow the Xanthomonas.org nomenclature.

| **Effectors** | **ACCESSION (NCBI)** |
| --- | --- |
| AvrBs2 | AAM34968.1 |
| AvrXccA1 | AAM43445.1 |
| AvrXccA2 | AAM41674.1 |
| HpaA | AAM35291.1 |
| HrpW | AAM37767.1 |
| XopA | AAM35307.1 |
| XopAA | CAJ25516.2 |
| XopAB | BAE69905.1 |
| XopAC | AAM41837.1 |
| XopAD | AAM39048.1 |
| XopAE | AAM35284.1 |
| XopAF1 | ZP_02241516.1 |
| XopAF2 | AEQ94681 |
| XopAG | AAM42870.1 |
| XopAH | AAM41397.1 |
| XopAI | AAM38074.1 |
| XopAJ | CAJ26159.1 |
| XopAK | AAM38509.1 |
| XopAL1 | AAM40544.1 |
| XopAL2 | CAP49952.1 |
| XopAM | AAM40388.1 |
| XopAP | AAM37835.1 |
| XopAU | AAM36043.1 |
| XopAV | AAM36044 |
| XopAW | AAM37794.1 |
| XopAX | CAJ19898.1 |
| XopAY | AAM36044.1 |
| XopAZ | AAM36229.1 |
| XopB | CAJ22212.1 |
| XopC1 | CAJ24112.1 |
| XopC2 | AAM36082.1 |
| XopD | CAJ22068.1 |
| XopE1 | AAM35178.1 |
| XopE2 | AAM39257.1 |
| XopE3 | AAM38068.1 |
| XopF1 | CAJ22045.1 |
| XopF2 | AAM37630.1 |
| XopG1 | CAJ22929.1 |
| XopG2 | AAM42528.1 |
| XopH1 | CAJ19917.1 |
| XopI1 | AAM35643.1 |
| XopJ1 | CAJ23833.1 |
| XopJ2 | ABM32744.1 |
| XopJ3 | CAJ22102.1 |
| XopJ4 | CAD14528.1 |
| XopJ5 | AAM42989.1 |
| XopK | AAM37930.1 |
| XopL | AAM37935.1 |
| XopM | AAM35309.1 |
| XopN | AAM37631.1 |
| XopO | CAJ22686.1 |
| XopP | AAM36080.1 |
| XopQ | AAM39163.1 |
| XopR | AAM35169.1 |
| XopS | AAM35207.1 |
| XopT | ABJ89951.1 |
| XopU | BAE69632.1 |
| XopV | AAM35490.1 |
| XopW | BAE66792.1 |
| XopX | AAM35432.1 |
| XopY | BAE68243.1 |
| XopZ1 | AAM36871.1 |
