# Supplementary material for: Analyses of Seven New Genomes of Xanthomonas citri pv. aurantifolii Strains, Causative Agents of Citrus Canker B and C, Show a Reduced Repertoire of Pathogenicity-Related Genes
Source: Front Microbiol. 2019 Oct 11;10:2361. doi: 10.3389/fmicb.2019.02361 (PMC6797930; doi:10.3389/fmicb.2019.02361)
Supplement: TABLE S2 — Representation of an alignment of ortholog genes having as anchor the Xac306 genome. [file Table_2.DOCX]

**Table ST2:** Representation of an alignment of ortholog genes having as anchor the Xac306 genome. Colored cells mean that the gene is present and cells with no color mean that the gene is absent.

|  | Xac306 | XauB11122 | XauB1561 | XauB1566 | XauC10535 | XauC1559 | XauC1609 | XauC535 | XauC763 | XauC867 | Xfus4834 | Product |
| --- | --- | --- | --- | --- | --- | --- | --- | --- | --- | --- | --- | --- |
| T1SS | +XAC3463 |  |  |  |  |  |  |  |  |  |  | [806] TolC protein |
|  | -XAC2201 |  |  |  |  |  |  |  |  |  |  | [3762] hemolysin secretion protein D |
|  | -XAC2202 |  |  |  |  |  |  |  |  |  |  | [3744] hemolysin secretion protein B |
|  |  |  |  |  |  |  |  |  |  |  |  |  |
| T2SS | +XAC0694 |  |  |  |  |  |  |  |  |  |  | [2250] type II secretion system protein C |
|  | +XAC0695 |  |  |  |  |  |  |  |  |  |  | [2249] type II secretion system protein D |
|  | +XAC0696 |  |  |  |  |  |  |  |  |  |  | [2248] type II secretion system protein E |
|  | +XAC0697 |  |  |  |  |  |  |  |  |  |  | [2247] type II secretion system protein F |
|  | +XAC0698 |  |  |  |  |  |  |  |  |  |  | [2246] type II secretion system protein G |
|  | +XAC0699 |  |  |  |  |  |  |  |  |  |  | [2245] type II secretion system protein H |
|  | +XAC0700 |  |  |  |  |  |  |  |  |  |  | [2244] type II secretion system protein I |
|  | +XAC0701 |  |  |  |  |  |  |  |  |  |  | [2243] type II secretion system protein J |
|  | +XAC0702 |  |  |  |  |  |  |  |  |  |  | [2242] type II secretion system protein K |
|  | +XAC0703 |  |  |  |  |  |  |  |  |  |  | [2241] type II secretion system protein L |
|  | +XAC0704 |  |  |  |  |  |  |  |  |  |  | [2240] type II secretion system protein M |
|  | +XAC0705 |  |  |  |  |  |  |  |  |  |  | [2239] type II secretion system protein N |
|  |  |  |  |  |  |  |  |  |  |  |  |  |
| T2SS | -XAC3534 |  |  |  |  |  |  |  |  |  |  | [765] general secretion pathway protein D |
|  | -XAC3535 |  |  |  |  |  |  |  |  |  |  | [764] general secretion pathway protein N |
|  | -XAC3536 |  |  |  |  |  |  |  |  |  |  | [763] general secretion pathway protein M |
|  | -XAC3537 |  |  |  |  |  |  |  |  |  |  | [762] general secretion pathway protein L |
|  | -XAC3538 |  |  |  |  |  |  |  |  |  |  | [761] general secretion pathway protein K |
|  | -XAC3539 |  |  |  |  |  |  |  |  |  |  | [2724] general secretion pathway protein J |
|  | -XAC3540 |  |  |  |  |  |  |  |  |  |  | [760] general secretion pathway protein I |
|  | -XAC3541 |  |  |  |  |  |  |  |  |  |  | [759] general secretion pathway protein H |
|  | -XAC3542 |  |  |  |  |  |  |  |  |  |  | [758] general secretion pathway protein G |
|  | -XAC3543 |  |  |  |  |  |  |  |  |  |  | [757] general secretion pathway protein F |
|  | -XAC3544 |  |  |  |  |  |  |  |  |  |  | [756] general secretion pathway protein |

|  |  |  |  |  |  |  |  |  |  |  |  |  |
| --- | --- | --- | --- | --- | --- | --- | --- | --- | --- | --- | --- | --- |
| T3SS | -XAC0393 |  |  |  |  |  |  |  |  |  |  | [3582] HpaF protein |
|  | -XAC0394 |  |  |  |  |  |  |  |  |  |  | [2427] HrpF protein |
|  | -XAC0395 |  |  |  |  |  |  |  |  |  |  | [singleton] hypothetical protein |
|  | -XAC0396 |  |  |  |  |  |  |  |  |  |  | [2426] HpaB protein |
|  | -XAC0397 |  |  |  |  |  |  |  |  |  |  | [3259] HrpE protein |
|  | -XAC0398 |  |  |  |  |  |  |  |  |  |  | [3258] HrpD6 protein |
|  | -XAC0399 |  |  |  |  |  |  |  |  |  |  | [2425] HrpD5 protein |
|  | -XAC0400 |  |  |  |  |  |  |  |  |  |  | [2424] HpaA protein |
|  | -XAC0401 |  |  |  |  |  |  |  |  |  |  | [3257] HrcS protein |
|  | -XAC0402 |  |  |  |  |  |  |  |  |  |  | [2423] type III secretion system protein |
|  | -XAC0403 |  |  |  |  |  |  |  |  |  |  | [2422] HrcQ protein |
|  | -XAC0404 |  |  |  |  |  |  |  |  |  |  | [2421] HpaP protein |
|  | -XAC0405 |  |  |  |  |  |  |  |  |  |  | [2420] HrcV protein |
|  | -XAC0406 |  |  |  |  |  |  |  |  |  |  | [2419] type III secretion system protein HrcU |
|  | +XAC0407 |  |  |  |  |  |  |  |  |  |  | [2418] HrpB1 protein |
|  | +XAC0408 |  |  |  |  |  |  |  |  |  |  | [2417] HrpB2 protein |
|  | +XAC0409 |  |  |  |  |  |  |  |  |  |  | [2416] HrcJ protein |
|  | +XAC0410 |  |  |  |  |  |  |  |  |  |  | [2415] HrpB4 protein |
|  | +XAC0411 |  |  |  |  |  |  |  |  |  |  | [2414] type III secretion system protein HrpB |
|  | +XAC0412 |  |  |  |  |  |  |  |  |  |  | [2413] type III secretion system ATPase |
|  | +XAC0413 |  |  |  |  |  |  |  |  |  |  | [2412] HrpB7 protein |
|  | +XAC0414 |  |  |  |  |  |  |  |  |  |  | [2411] HrcT protein |
|  | +XAC0415 |  |  |  |  |  |  |  |  |  |  | [2410] HrcC protein |
|  | -XAC0416 |  |  |  |  |  |  |  |  |  |  | [2409] Hpa1 protein |
|  | +XAC0417 |  |  |  |  |  |  |  |  |  |  | [295] Hpa2 protein |
|  | -XAC1265 |  |  |  |  |  |  |  |  |  |  | [1008] HrpG protein |
|  | +XAC1266 |  |  |  |  |  |  |  |  |  |  | [1009] HrpX protein |
|  | -XAC1994 |  |  |  |  |  |  |  |  |  |  | [1432] HrpX-like protein |
|  | +XAC2922 |  |  |  |  |  |  |  |  |  |  | [3729] HrpW protein |

|  |  |  |  |  |  |  |  |  |  |  |  |  |
| --- | --- | --- | --- | --- | --- | --- | --- | --- | --- | --- | --- | --- |
| T4SS_P | -XACb0036 |  |  |  |  |  |  |  |  |  |  | [3741] VirB1 protein |
|  | -XACb0037 |  |  |  |  |  |  |  |  |  |  | [3787] VirB11 protein |
|  | -XACb0038 |  |  |  |  |  |  |  |  |  |  | [3771] VirB10 protein |
|  | -XACb0039 |  |  |  |  |  |  |  |  |  |  | [3770] VirB9 protein |
|  | - |  |  |  |  |  |  |  |  |  |  |  |
|  | -XACb0040 |  |  |  |  |  |  |  |  |  |  | [3786] VirB8 protein |
|  | -XACb0041 |  |  |  |  |  |  |  |  |  |  | [3752] VirB6 protein |
|  | -XACb0042 |  |  |  |  |  |  |  |  |  |  | [3769] hypothetical protein |
|  | -XACb0043 |  |  |  |  |  |  |  |  |  |  | [3875] hypothetical protein |
|  | -XACb0044 |  |  |  |  |  |  |  |  |  |  | [3781] VirB5 protein |
|  | -XACb0045 |  |  |  |  |  |  |  |  |  |  | [3768] VirB4 protein |
|  | - |  |  |  |  |  |  |  |  |  |  |  |
|  | -XACb0046 |  |  |  |  |  |  |  |  |  |  | [3820] VirB3 protein |
|  | - |  |  |  |  |  |  |  |  |  |  |  |
|  | -XACb0047 |  |  |  |  |  |  |  |  |  |  | [3751] VirB2 protein |
|  | - |  |  |  |  |  |  |  |  |  |  |  |
|  |  |  |  |  |  |  |  |  |  |  |  |  |
| T4SS_C | -XAC2607 |  |  |  |  |  |  |  |  |  |  | [singleton] VirB6 protein |
|  | -XAC2608 |  |  |  |  |  |  |  |  |  |  | [singleton] VirB6 protein |
|  | -XAC2609 |  |  |  |  |  |  |  |  |  |  | [3503] carboxypeptidase |
|  | -XAC2610 |  |  |  |  |  |  |  |  |  |  | [3807] hypothetical protein |
|  | -XAC2611 |  |  |  |  |  |  |  |  |  |  | [5202] hypothetical protein |
|  | -XAC2612 |  |  |  |  |  |  |  |  |  |  | [3392] VirB6 protein |
|  | -XAC2613 |  |  |  |  |  |  |  |  |  |  | [3808] hypothetical protein |
|  | -XAC2614 |  |  |  |  |  |  |  |  |  |  | [3393] VirB4 protein |
|  | -XAC2615 |  |  |  |  |  |  |  |  |  |  | [3748] VirB3 protein |
|  | -XAC2616 |  |  |  |  |  |  |  |  |  |  | [3723] VirB2 protein |
|  | -XAC2617 |  |  |  |  |  |  |  |  |  |  | [3724] VirB1 protein |
|  | -XAC2618 |  |  |  |  |  |  |  |  |  |  | [3504] VirB11 protein |
|  | -XAC2619 |  |  |  |  |  |  |  |  |  |  | [3764] VirB10 protein |
|  | -XAC2620 |  |  |  |  |  |  |  |  |  |  | [3613] VirB9 protein |
|  | -XAC2621 |  |  |  |  |  |  |  |  |  |  | [3699] VirB8 protein |
|  | -XAC2622 |  |  |  |  |  |  |  |  |  |  | [3505] hypothetical protein |
|  | -XAC2623 |  |  |  |  |  |  |  |  |  |  | [92] VirD4 protein |
|  | - |  |  |  |  |  |  |  |  |  |  |  |

| T4p | +XAC3238 |  |  |  |  |  |  |  |  |  |  | [1782] sensor histidine kinase PilS |
| --- | --- | --- | --- | --- | --- | --- | --- | --- | --- | --- | --- | --- |
|  | +XAC3239 |  |  |  |  |  |  |  |  |  |  | [3369] sigma-54-dependent Fis family transcriptional regulator PilR |
|  | -XAC3239 |  |  |  |  |  |  |  |  |  |  | [3185] type IV-A pilus assembly ATPase PilB |
|  | -XAC3240 |  |  |  |  |  |  |  |  |  |  | [singleton]] pilin PilA |
|  | -XAC3241 |  |  |  |  |  |  |  |  |  |  | [singleton] prepilin-type cleavage/methylation domain-containing protein PilA |
|  | +XAC3242 |  |  |  |  |  |  |  |  |  |  | [2861] type II secretion system F family protein PilC |
|  | +XAC3243 |  |  |  |  |  |  |  |  |  |  | [2860] prepilin peptidase PilD |
|  | -XAC2664 |  |  |  |  |  |  |  |  |  |  | [2937] type IV pilin protein PilE |
|  | -XAC2665 |  |  |  |  |  |  |  |  |  |  | [2936] pilus assembly protein PilY1 |
|  | -XAC2666 |  |  |  |  |  |  |  |  |  |  | [singleton] pilus assembly protein PilX |
|  | -XAC2667 |  |  |  |  |  |  |  |  |  |  | [singleton] prepilin-type cleavage/methylation domain-containing protein PilW |
|  | -XAC2668 |  |  |  |  |  |  |  |  |  |  | [2598] type IV pilus modification protein PilV |
|  | -XAC2669 |  |  |  |  |  |  |  |  |  |  | [90] prepilin-type N-terminal cleavage/methylation domain-containing protein FimT |
|  | -XAC2670 |  |  |  |  |  |  |  |  |  |  | [singleton] sensor histidine kinase / alginate biosynthesis protein |
|  | -XAC2924 |  |  |  |  |  |  |  |  |  |  | [1398] twitching motility protein PilT |
|  | -XAC2923 |  |  |  |  |  |  |  |  |  |  | [1399] type IV pili twitching motility protein PilU |
|  | -XAC3381 |  |  |  |  |  |  |  |  |  |  | [3119] type IV pilus secretin PilQ |
|  | -XAC3382 |  |  |  |  |  |  |  |  |  |  | [762] type IV pilus assembly protein PilP |
|  | -XAC3383 |  |  |  |  |  |  |  |  |  |  | [761] fimbrial protein PilO |
|  | -XAC3384 |  |  |  |  |  |  |  |  |  |  | [760] fimbrial protein PilN |
|  | -XAC3385 |  |  |  |  |  |  |  |  |  |  | [759] pilus assembly protein PilM |
|  |  |  |  |  |  |  |  |  |  |  |  |  |
| SEC | -XAC2510 |  |  |  |  |  |  |  |  |  |  | [1625] preprotein translocase subunit SecF |
|  | -XAC2511 |  |  |  |  |  |  |  |  |  |  | [1626] preprotein translocase subunit SecD |
|  | -XAC2512 |  |  |  |  |  |  |  |  |  |  | [1627] preprotein translocase subunit YajC |
|  | +XAC0959 |  |  |  |  |  |  |  |  |  |  | [2092] preprotein translocase subunit SecE |
|  | +XAC0992 |  |  |  |  |  |  |  |  |  |  | [2069] preprotein translocase subunit SecY |
|  | -XAC2706 |  |  |  |  |  |  |  |  |  |  | [2647] preprotein translocase subunit SecG |
|  | -XAC4372 |  |  |  |  |  |  |  |  |  |  | [330] inner membrane protein translocase component YidC |
|  | +XAC0788 |  |  |  |  |  |  |  |  |  |  | [2187] preprotein translocase subunit SecA |
|  | +XAC2552 |  |  |  |  |  |  |  |  |  |  | [1654] cell division protein |
|  | +XAC0221 |  |  |  |  |  |  |  |  |  |  | [2539] preprotein translocase subunit SecB |
|  | +XAC1289 |  |  |  |  |  |  |  |  |  |  | [1025] signal recognition particle protein |
|  |  |  |  |  |  |  |  |  |  |  |  |  |
| TAT | -XAC4216 |  |  |  |  |  |  |  |  |  |  | [399] sec-independent protein translocase |
|  | -XAC4217 |  |  |  |  |  |  |  |  |  |  | [398] sec-independent translocase |
|  | -XAC4218 |  |  |  |  |  |  |  |  |  |  | [3032] twin-arginine translocation protein TatA |
|  |  |  |  |  |  |  |  |  |  |  |  |  |
| T6SS | -XAC4124 |  |  |  |  |  |  |  |  |  |  | [89] hypothetical protein |
|  | XAC4145 |  |  |  |  |  |  |  |  |  |  | [436] hypothetical protein |
|  | -XAC4119 |  |  |  |  |  |  |  |  |  |  | [91] hypothetical protein |
|  | -XAC4120 |  |  |  |  |  |  |  |  |  |  | [448] hypothetical protein |
|  | -XAC4139 |  |  |  |  |  |  |  |  |  |  | [62] chaperone ClpB |
|  | -XAC4140 |  |  |  |  |  |  |  |  |  |  |  |

| Gum Cluster | -XAC2570 |  |  |  |  |  |  |  |  |  |  | [1665] GumP protein |
| --- | --- | --- | --- | --- | --- | --- | --- | --- | --- | --- | --- | --- |
|  | -XAC2571 |  |  |  |  |  |  |  |  |  |  | [2879] 3-oxoacyl-ACP synthase |
|  | -XAC2572 |  |  |  |  |  |  |  |  |  |  | [2880] GumN protein |
|  | -XAC2573 |  |  |  |  |  |  |  |  |  |  | [1666] hypothetical protein |
|  | -XAC2574 |  |  |  |  |  |  |  |  |  |  | [1667] GumM protein |
|  | -XAC2575 |  |  |  |  |  |  |  |  |  |  | [1668] GumL protein |
|  | -XAC2576 |  |  |  |  |  |  |  |  |  |  | [1669] GumK protein |
|  | -XAC2577 |  |  |  |  |  |  |  |  |  |  | [1670] GumJ protein |
|  | -XAC2578 |  |  |  |  |  |  |  |  |  |  | [1671] GumI protein |
|  | -XAC2579 |  |  |  |  |  |  |  |  |  |  | [1672] GumH protein |
|  | -XAC2580 |  |  |  |  |  |  |  |  |  |  | [1673] GumG protein |
|  | -XAC2581 |  |  |  |  |  |  |  |  |  |  | [1674] GumF protein |
|  | -XAC2582 |  |  |  |  |  |  |  |  |  |  | [238] GumE protein |
|  | - |  |  |  |  |  |  |  |  |  |  |  |
|  | -XAC2583 |  |  |  |  |  |  |  |  |  |  | [1675] GumD protein |
|  | -XAC2584 |  |  |  |  |  |  |  |  |  |  | [1676] GumC protein |
|  | -XAC2585 |  |  |  |  |  |  |  |  |  |  | [1677] GumB protein |
|  |  |  |  |  |  |  |  |  |  |  |  |  |
| rpf_cluster | +XAC1864 |  |  |  |  |  |  |  |  |  |  | [1389] regulatory protein |
|  | +XAC1865 |  |  |  |  |  |  |  |  |  |  | [1390] single-stranded-DNA-specific exonuclease |
|  | +XAC1304 |  |  |  |  |  |  |  |  |  |  | [singleton] wall associated protein |
|  | +XAC1866 |  |  |  |  |  |  |  |  |  |  |  |
|  | +XAC1867 |  |  |  |  |  |  |  |  |  |  | [singleton] hypothetical protein |
|  | +XAC1868 |  |  |  |  |  |  |  |  |  |  | [singleton] hypothetical protein |
|  | +XAC1869 |  |  |  |  |  |  |  |  |  |  | [singleton] hypothetical protein |
|  | +XAC1870 |  |  |  |  |  |  |  |  |  |  | [singleton] hypothetical protein |
|  | -XAC1871 |  |  |  |  |  |  |  |  |  |  | [4433] transposase |
|  | -XAC2508 |  |  |  |  |  |  |  |  |  |  |  |
|  | -XAC1872 |  |  |  |  |  |  |  |  |  |  | [singleton] transposase |
|  | -XAC1873 |  |  |  |  |  |  |  |  |  |  | [1391] hypothetical protein |
|  | +XAC1874 |  |  |  |  |  |  |  |  |  |  | [3496] regulatory protein |
|  | +XAC1875 |  |  |  |  |  |  |  |  |  |  | [1392] peptide chain release factor 2 |
|  | +XAC1876 |  |  |  |  |  |  |  |  |  |  | [1393] lysyl-tRNA synthetase |
|  | +XAC1877 |  |  |  |  |  |  |  |  |  |  | [1394] response regulator |
|  | +XAC1878 |  |  |  |  |  |  |  |  |  |  | [1395] RpfC protein |
|  | -XAC1879 |  |  |  |  |  |  |  |  |  |  | [1396] enoyl-CoA hydratase |
|  | -XAC1880 |  |  |  |  |  |  |  |  |  |  | [2813] long-chain fatty acid-CoA ligase |
|  | +XAC1881 |  |  |  |  |  |  |  |  |  |  | [1397] hypothetical protein |
|  | +XAC1139 |  |  |  |  |  |  |  |  |  |  | [28] aconitate hydratase |
|  | -XAC1882 |  |  |  |  |  |  |  |  |  |  |  |

|  |  |  |  |  |  |  |  |  |  |  |  |  |
| --- | --- | --- | --- | --- | --- | --- | --- | --- | --- | --- | --- | --- |
| LPS cluster I | -XAC3577 |  |  |  |  |  |  |  |  |  |  | [734] IpsJ protein |
|  | -XAC3578 |  |  |  |  |  |  |  |  |  |  | [733] IpsJ protein |
|  | +XAC3579 |  |  |  |  |  |  |  |  |  |  | [732] phosphoglucomutase |
|  | +XAC3580 |  |  |  |  |  |  |  |  |  |  | [731] GDP-mannose pyrophosphorylase |
|  | -XAC3581 |  |  |  |  |  |  |  |  |  |  | [730] UDP-glucose dehydrogenase |
|  | -XAC3582 |  |  |  |  |  |  |  |  |  |  | [729] dTDP-4-keto-L-rhamnose reductase |
|  | -XAC3583 |  |  |  |  |  |  |  |  |  |  | [728] dTDP-4-dehydrorhamnose 3,5-epimerase |
|  | -XAC3584 |  |  |  |  |  |  |  |  |  |  | [727] glucose-1-phosphate thymidylyltransferase |
|  | -XAC3585 |  |  |  |  |  |  |  |  |  |  | [726] dTDP-glucose 4,6-dehydratase |
|  | +XAC3586 |  |  |  |  |  |  |  |  |  |  | [725] electron transfer flavoprotein subunit beta |
|  | +XAC3587 |  |  |  |  |  |  |  |  |  |  | [724] electron transfer flavoprotein alpha subunit |
|  | +XAC3588 |  |  |  |  |  |  |  |  |  |  | [3473] hypothetical protein |
|  | +XAC3589 |  |  |  |  |  |  |  |  |  |  | [3404] hypothetical protein |
|  |  |  |  |  |  |  |  |  |  |  |  |  |
|  | +XAC3590 |  |  |  |  |  |  |  |  |  |  | [3469] oxidoreductase |
|  | +XAC3591 |  |  |  |  |  |  |  |  |  |  | [3472] short chain dehydrogenase |
|  | +XAC3592 |  |  |  |  |  |  |  |  |  |  | [singleton] hypothetical protein |
|  | +XAC3593 |  |  |  |  |  |  |  |  |  |  | [3471] NAD dependent epimerase/dehydratase/dehydrogenase |
|  | +XAC3594 |  |  |  |  |  |  |  |  |  |  | [723] hypothetical protein |
|  |  |  |  |  |  |  |  |  |  |  |  |  |
|  | +XAC3595 |  |  |  |  |  |  |  |  |  |  | [3470] hypothetical protein |
|  | +XAC3596 |  |  |  |  |  |  |  |  |  |  | [singleton] hypothetical protein |
|  | -XAC3597 |  |  |  |  |  |  |  |  |  |  | [singleton] hypothetical protein |
|  | -XAC3598 |  |  |  |  |  |  |  |  |  |  | [singleton] hypothetical protein |
|  | -XAC3599 |  |  |  |  |  |  |  |  |  |  | [singleton] hypothetical protein |
|  | -XAC3600 |  |  |  |  |  |  |  |  |  |  | [722] ABC transporter ATP-binding protein |
|  | -XAC3601 |  |  |  |  |  |  |  |  |  |  | [3288] ABC transporter permease |
|  | -XAC3602 |  |  |  |  |  |  |  |  |  |  | [721] cystathionine gamma-synthase |
|  | -XAC3603 |  |  |  |  |  |  |  |  |  |  | [720] cystathionine beta-synthase |
|  | -XAC3604 |  |  |  |  |  |  |  |  |  |  | [3287] hypothetical protein |
|  | -XAC3605 |  |  |  |  |  |  |  |  |  |  | [719] hypothetical protein |
|  | -XAC3606 |  |  |  |  |  |  |  |  |  |  | [718] hypothetical protein |

|  |  |  |  |  |  |  |  |  |  |  |  |  |
| --- | --- | --- | --- | --- | --- | --- | --- | --- | --- | --- | --- | --- |
| LPS cluster II | +XAC0037 |  |  |  |  |  |  |  |  |  |  | [singleton] penicillin acylase |
|  |  |  |  |  |  |  |  |  |  |  |  |  |
|  | -XAC0038 |  |  |  |  |  |  |  |  |  |  | [singleton] hypothetical protein |
|  | +XAC0039 |  |  |  |  |  |  |  |  |  |  | [singleton] transcriptional regulator |
|  | -XAC0040( |  |  |  |  |  |  |  |  |  |  | [singleton] hypothetical protein |
|  | +XAC0041 |  |  |  |  |  |  |  |  |  |  | [singleton] mannosyltransferase |
|  | +XAC0042 |  |  |  |  |  |  |  |  |  |  | [singleton] glycosyltransferase |
|  | +XAC0043 |  |  |  |  |  |  |  |  |  |  | [singleton] UDP-glucose lipid carrier transferase |
|  | +XAC0044 |  |  |  |  |  |  |  |  |  |  | [singleton] lipopolysaccharide biosynthesis protein |
|  | -XAC0045 |  |  |  |  |  |  |  |  |  |  | [singleton] hypothetical protein |
|  | -XAC0046 |  |  |  |  |  |  |  |  |  |  | [singleton] UDP-N-acetyl-D-mannosamine transferase |
|  | -XAC0047 |  |  |  |  |  |  |  |  |  |  | [singleton] galactosyltransferase |
|  | -XAC0048 |  |  |  |  |  |  |  |  |  |  | [singleton] hypothetical protein |
|  | -XAC0049 |  |  |  |  |  |  |  |  |  |  | [singleton] hypothetical protein |
|  | -XAC0050 |  |  |  |  |  |  |  |  |  |  | [singleton] hypothetical protein |
|  | -XAC0051 |  |  |  |  |  |  |  |  |  |  | [singleton] asparagine synthase |
|  | -XAC0052 |  |  |  |  |  |  |  |  |  |  | [singleton] hypothetical protein |
|  | -XAC0053 |  |  |  |  |  |  |  |  |  |  | [singleton] methyltransferase |
|  | -XAC0054 |  |  |  |  |  |  |  |  |  |  | [singleton] degenerated UDP-glucose epimerase |
|  | -XAC0055 |  |  |  |  |  |  |  |  |  |  | [singleton] hypothetical protein |
|  | -XAC0056 |  |  |  |  |  |  |  |  |  |  | [singleton] polysaccharide export protein |
|  | -XAC0057 |  |  |  |  |  |  |  |  |  |  | [singleton] transporter |
|  | -XAC0058 |  |  |  |  |  |  |  |  |  |  | [singleton] hypothetical protein |
|  | -XAC0059 |  |  |  |  |  |  |  |  |  |  | [singleton] asparagine synthetase like protein |
|  | +XAC0060 |  |  |  |  |  |  |  |  |  |  | [singleton] hypothetical protein |
|  | +XAC0061 |  |  |  |  |  |  |  |  |  |  | [singleton] hypothetical protein |
|  | +XAC0062 |  |  |  |  |  |  |  |  |  |  | [singleton] hypothetical protein |
|  | +XAC0063 |  |  |  |  |  |  |  |  |  |  | [singleton] arylsulfotransferase |
|  | -XAC0064 |  |  |  |  |  |  |  |  |  |  | [3468] acetyltransferase |
|  | -XAC0065 |  |  |  |  |  |  |  |  |  |  | [singleton] microcystin dependent protein |
|  | -XAC0066 |  |  |  |  |  |  |  |  |  |  | [2620] microcystin dependent protein |
|  | -XAC0067 |  |  |  |  |  |  |  |  |  |  | [2619] microcystin dependent protein |
|  | +XAC0068 |  |  |  |  |  |  |  |  |  |  | [singleton] hypothetical protein |

|  |  |  |  |  |  |  |  |  |  |  |  |  |
| --- | --- | --- | --- | --- | --- | --- | --- | --- | --- | --- | --- | --- |
| Xanthomonadin | XAC4084 |  |  |  |  |  |  |  |  |  |  | [2686] ankyrin-like protein |
|  | -XAC4085 |  |  |  |  |  |  |  |  |  |  | [3357] hypothetical protein |
|  | +XAC4086 |  |  |  |  |  |  |  |  |  |  | [462] 3-oxoacyl-ACP synthase |
|  | +XAC4087 |  |  |  |  |  |  |  |  |  |  | [3045] hypothetical protein |
|  | +XAC4088 |  |  |  |  |  |  |  |  |  |  | [461] dolichyl-phosphate mannose synthase-like protein |
|  | +XAC4089 |  |  |  |  |  |  |  |  |  |  | [2685] halogenase |
|  | -XAC4090 |  |  |  |  |  |  |  |  |  |  | [2684] 3-ketoacyl-ACP reductase |
|  | +XAC4091 |  |  |  |  |  |  |  |  |  |  | [3044] hypothetical protein |
|  | -XAC4092 |  |  |  |  |  |  |  |  |  |  | [2683] phosphotransferase |
|  | -XAC4093 |  |  |  |  |  |  |  |  |  |  | [3043] hypothetical protein |
|  | -XAC4094 |  |  |  |  |  |  |  |  |  |  | [78] hypothetical protein |
|  | -XAC4095 |  |  |  |  |  |  |  |  |  |  |  |
|  | -XAC4094 |  |  |  |  |  |  |  |  |  |  | [78] hypothetical protein |
|  | -XAC4095 |  |  |  |  |  |  |  |  |  |  |  |
|  | -XAC4096 |  |  |  |  |  |  |  |  |  |  | [2682] fatty acyl CoA synthetase |
|  | -XAC4097 |  |  |  |  |  |  |  |  |  |  | [2681] acyltransferase |
|  | -XAC4098 |  |  |  |  |  |  |  |  |  |  | [3281] dehydratase |
|  | -XAC4099 |  |  |  |  |  |  |  |  |  |  | [2680] acyltransferase |
|  | -XAC4100 |  |  |  |  |  |  |  |  |  |  | [460] ketosynthase |
|  | -XAC4101 |  |  |  |  |  |  |  |  |  |  | [459] acyl carrier protein |
|  | +XAC4102 |  |  |  |  |  |  |  |  |  |  | [458] hydroxylase |
|  | +XAC4103 |  |  |  |  |  |  |  |  |  |  | [457] pteridine-dependent deoxygenase like protein |
|  | +XAC4104 |  |  |  |  |  |  |  |  |  |  | [3537] hypothetical protein |
|  | -XAC4105 |  |  |  |  |  |  |  |  |  |  | [456] AMP-ligase |
|  | +XAC4106 |  |  |  |  |  |  |  |  |  |  | [455] dipeptidyl peptidase |
|  | +XAC4107 |  |  |  |  |  |  |  |  |  |  | [454] hypothetical protein |
|  | +XAC4108 |  |  |  |  |  |  |  |  |  |  | [453] hypothetical protein |
