# Supplementary material for: Analyses of Seven New Genomes of Xanthomonas citri pv. aurantifolii Strains, Causative Agents of Citrus Canker B and C, Show a Reduced Repertoire of Pathogenicity-Related Genes
Source: Front Microbiol. 2019 Oct 11;10:2361. doi: 10.3389/fmicb.2019.02361 (PMC6797930; doi:10.3389/fmicb.2019.02361)
Supplement: TABLE S3 — Results of biochemical assays related to xanthan gum and biofilm production, and aggregation (left-had side). [file Table_3.DOCX]

**Table ST3:** Results of biochemical assays related to xanthan gum and biofilm production, and aggregation (left-had side). Genes and gene clusters related to xanthan gum, biofilm and aggregation (right-hand side).

| **Strain** | **Biochemical assays** | | | | | | | | **Genes/clusters related to xanthan gum, biofilm and aggregation** | | | | | | | | | | | | | | | | |
| --- | --- | --- | --- | --- | --- | --- | --- | --- | --- | --- | --- | --- | --- | --- | --- | --- | --- | --- | --- | --- | --- | --- | --- | --- | --- |
|  | **Xanthan gum production** | | | | **Biofilm production** | | **Aggregation (12 h)** | |  |  |  |  |  |  |  |  |  |  |  |  |  |  |  |  |  |
|  | **Bac mg/mL (SD)** | **Gum mg/mL (SD)** | **Gum/Bac (mg/mg)** | **%*** | **LB Abs (SD)** | **XVM2 Abs (SD)** | **LB (%)** | **XVM2 (%)** | ***Gum/rpf* clusters** | **ax21** | **Cyclic-di-GMP** | | | | | **Fimbrillin genes** | **Pili/fimbrial genes** | **T4SS** | **Hms cluster** | **Adhesins**  ***xad/yapH*** | **LPS cluster (*xanA/xanB/ugd*)** | **O-antigen clusters** | ***rsmA*/*csrA/clp*** | ***rpfN*/*/ompW*** | **TBDR** |
|  |  |  |  |  |  |  |  |  |  |  | **GGDEF** | | **EAL** | **HD-GYP** | **PilZ** |  |  |  |  |  |  |  |  |  |  |
| A306 | 1.35 (±0.0) | 3.19 (±0.48) | 2.36 (±0.25) | 100.0 | 0.10 (±0.01) | 0.13 (±0.02) | 4.92 (±0.01) | 79.63 (±0.01) | C/C | + | 29 | 14 | | 3 | 3 | 3 | 15 | Ch/Pl | C | 2/+ | C | I-II | +/+/+ | +/+ | 46 |
| XauB1566 | 1.30 (±0.8) | 2.03 (±0.43) | 1.24 (±0.13) | 59.0 | 0.15 (±0.04) | 0.10 (±0.02) | 30.53 (±0.06) | 62.54 (±0.05) | C/C | + | 23 | 13 | | 3 | 4 | 1 | 9 | Pl | NF | 0/- | C | I | +/+/+ | -/+ | 45 |
| XauB1561 | 1.41 (±0.10) | 2.12 (±0.57) | 1.49 (±0.26) | 63.0 | 0.17 (±0.08) | 0.12 (±0.02) | 36.78 (±0.03) | 67.87 (±0.00) | C/C | - | 25 | 14 | | 3 | 4 | 1 | 9 | Pl | NF | 0/- | C | I | +/+/+ | -/+ | 46 |
| XauC535 | 2.09 (±0.08) | 1.36 (±0.06) | 0.65 (±0.03) | 27.4 | 0.17 (±0.06) | 0.16 (±0.03) | 5.30 (±0.00) | 68.16 (±0.00) | C/C | + | 28 | 15 | | 3 | 4 | 1 | 10 | Pl | NF | 3/+ | C | I | +/+/+ | +/+ | 44 |
| XauC763 | 1.80 (±0.06) | 1.08 (±0.09) | 0.60 (±0.02) | 25.4 | 0.15 (±0.03) | 0.10 (±0.01) | 4.53 (±0.02) | 71.17 (±0.01) | C/C | + | 29 | 14 | | 3 | 4 | 1 | 10 | Pl | NF | 1/+ | C | I | +/+/+ | +/+ | 45 |
| XauC1609 | 1.83 (±0.08) | 0.92 (±0.08) | 0.50 (±0.05) | 21.3 | 0.11 (±0.01) | 0.11 (±0.02) | 16.36 (±0.01) | 100 (±0.00) | C/C | + | 28 | 14 | | 3 | 4 | 1 | 10 | Pl | NF | 2/+ | C | I | +/+/+ | +/+ | 43 |
| XauC1559 | 1.90 (±0.14) | 0.79 (±0.08) | 0.42 (±0.04) | 17.6 | 0.10 (±0.00) | 0.17 (±0.02) | 9.88 (±0.00) | 81.86 (±0.00) | C/C | + | 29 | 13 | | 3 | 4 | 1 | 9 | Pl | NF | 1/+ | C | I | +/+/+ | +/+ | 42 |
| XauC867 | 1.78 (±0.22) | 0.51 (±0.10) | 0.28 (±0.02) | 11.9 | 0.10 (±0.01) | 0.11 (±0.02) | 82.94 (±0.01) | 70.53 (±0.00) | C/C | + | 29 | 14 | | 3 | 4 | 1 | 10 | Pl | NF | 1/+ | C | I | +/+/+ | +/+ | 43 |

Abs – Absorbance OD550 nm; SD – Standard deviation; Bac – bacteria; * related to XAC306; C – complete; NF – Not found, Ch – Chromosomal copy; Pl – plasmidial copy; “+” - Present; “-“ – Absent
